# Spatially Adjacent Regions in Posterior Cingulate Cortex Represent Familiar Faces at Different Levels of Complexity

**DOI:** 10.1523/JNEUROSCI.1580-20.2021

**Published:** 2021-11-24

**Authors:** Neda Afzalian, Reza Rajimehr

**Affiliations:** ^1^School of Cognitive Sciences, Institute for Research in Fundamental Sciences (IPM), Tehran, Iran 1956836484; ^2^MRC Cognition and Brain Sciences Unit, University of Cambridge, Cambridge, United Kingdom CB2 7EF; ^3^McGovern Institute for Brain Research, Massachusetts Institute of Technology, Cambridge, Massachusetts 02139

**Keywords:** face subcategories, familiar faces, fMRI, multivariate pattern analysis, posterior cingulate cortex, semantic processing

## Abstract

Extensive research has shown that perceptual information of faces is processed in a network of hierarchically-organized areas within ventral temporal cortex. For familiar and famous faces, perceptual processing of faces is normally accompanied by extraction of semantic knowledge about the social status of persons. Semantic processing of familiar faces could entail progressive stages of information abstraction. However, the cortical mechanisms supporting multistage processing of familiar faces have not been characterized. Here, using an event-related fMRI experiment, familiar faces from four celebrity groups (actors, singers, politicians, and football players) and unfamiliar faces were presented to the human subjects (both males and females) while they were engaged in a face categorization task. We systematically explored the cortical representations for faces, familiar faces, subcategories of familiar faces, and familiar face identities using whole-brain univariate analysis, searchlight-based multivariate pattern analysis (MVPA), and functional connectivity analysis. Convergent evidence from all these analyses revealed a set of overlapping regions within posterior cingulate cortex (PCC) that contained decodable fMRI responses for representing different levels of semantic knowledge about familiar faces. Our results suggest a multistage pathway in PCC for processing semantic information of faces, analogous to the multistage pathway in ventral temporal cortex for processing perceptual information of faces.

**SIGNIFICANCE STATEMENT** Recognizing familiar faces is an important component of social communications. Previous research has shown that a distributed network of brain areas is involved in processing the semantic information of familiar faces. However, it is not clear how different levels of semantic information are represented in the brain. Here, we evaluated the multivariate response patterns across the entire cortex to discover the areas that contain information for familiar faces, subcategories of familiar faces, and identities of familiar faces. The searchlight maps revealed that different levels of semantic information are represented in topographically adjacent areas within posterior cingulate cortex (PCC). The results suggest that semantic processing of faces is mediated through progressive stages of information abstraction in PCC.

## Introduction

Face recognition is a fundamental cognitive ability which plays an important role in social communications in everyday life. Faces, as complex visual stimuli, contain various types of information along different perceptual dimensions. One main goal of human visual system is to flexibly extract perceptual information of faces at multiple levels, including early visual processing for face detection, analysis of facial features and their configuration, analysis of facial viewpoints and expressions, and finally view-invariant processing for face identification. For familiar and famous faces, this cascade of perceptual analysis is followed by a process of retrieving semantic information or semantic knowledge about faces (Young et al., 1986). This process can be viewed as a continuum comprising multiple stages of information extraction, from the general facts about people to the specific facts about individuals. In light of such multistage abstraction of semantic information, familiar and famous faces can be processed hierarchically at different semantic levels: they can be processed as a general category of human faces, they can be categorized/distinguished from unfamiliar faces, they can be classified into specific subcategories of familiar faces based on social and contextual factors (e.g., celebrity groups such as actors and politicians), and they can be identified as unique individuals (e.g., the face of Brad Pitt or Barack Obama). Flexible and efficient extraction of face-related semantic information could be possible by having dedicated neural representations for every level of semantic processing. Although, previous studies have found hierarchical and multistage representations within ventral temporal cortex for perceptual processing of faces (Grill-Spector and Weiner, 2014), it remains an open question how and where in the brain multiple categorical levels of face-related semantic knowledge are represented, and whether there is a specific topographic organization for such representations.

Previous studies have shown that perceptual information of faces is processed by a distributed network of brain regions. These areas, which are considered as the core system of face processing, hierarchically transform perceptual information of individual face identity, either familiar or unfamiliar, from view-specific to identity-specific information (Haxby et al., 2000; Gobbini and Haxby, 2007). Indeed, perceptual information of faces elicits high activities in bilateral face-selective areas within occipitotemporal cortex: the occipital face area (OFA; Gauthier et al., 2000), the fusiform face area (FFA; Kanwisher et al., 1997), and the posterior superior temporal sulcus (pSTS; Chao et al., 1999; Grill-Spector and Weiner, 2014). Further research has reported that a distinct face-selective region in the ventral part of the anterior temporal cortex [named as anterior temporal face patch (ATFP); Rajimehr et al., 2009] is also involved in visual and perceptual encoding of faces. More specifically, ATFP appears to be an important region for recognition and discrimination of facial identities (Kriegeskorte et al., 2007; Nestor et al., 2011; Goesaert and Op de Beeck, 2013; Anzellotti et al., 2014).

While activity of posterior regions (OFA, FFA, and pSTS) revealed inconsistent and weak differences in response to cognitive manipulations such as familiarity (Dubois et al., 1999; Natu and O'Toole, 2011; Ramon and Gobbini, 2018), neural structures within anterior temporal lobe (ATL) exhibited enhanced activation to familiar/famous faces than unfamiliar faces (Gobbini et al., 2004; Gobbini and Haxby, 2007; Ramon et al., 2015). These structures also revealed an adaptation response to repetition of familiar faces (Sugiura et al., 2011) and showed sensitivity to association of semantic information to faces (Von Der Heide et al., 2013). Thus, it has been suggested that ATL is a well-suited area to bound perceptual information of faces with semantic knowledge of individuals including their names or biographical information (Collins and Olson, 2014).

Neuroimaging studies have also shown that beyond the ATL, a number of additional regions such as structures within posterior cingulate cortex (PCC), anterior cingulate cortex (ACC), medial temporal lobe (MTL), hippocampus, and amygdala are engaged in the processing of familiar/famous faces (Ramon et al., 2015; Weibert et al., 2016). These regions, which are located outside of classical visual areas, are part of a broad group of face patches defined as the extended system of face processing (Haxby et al., 2000; Gobbini and Haxby, 2007). The results from a meta-analysis also suggests that semantic information and person knowledge are mainly represented in the extended face network (Binder et al., 2009). These studies provide valuable insights into which areas of the brain contain the neural code for semantic knowledge of familiar faces. However, it still remains an open question whether and how those areas represent the continuum of person-related semantic knowledge, from general information about people to more abstract information about specific individuals.

In the study reported here, therefore, we aimed to address how semantic information of familiar faces is extracted in the human face processing system. To address this question, we needed an experimental paradigm to ensure that we target the neural structures that are involved in processing the fine-grained and abstract information of familiar faces with respect to social attributes. Thus, we designed an experiment to present multiple face identities from two main categories of familiar and unfamiliar faces, with familiar stimuli selected from four different subcategories of famous faces (four celebrity groups). This rich set of face stimuli provided us with the opportunity to investigate the neural structures representing and disentangling semantic information of faces in multiple levels of abstraction. The human subjects were engaged in a celebrity categorization task, and their brain responses were collected using an event-related fMRI experiment. We systematically increased the sensitivity of the analysis from univariate to multivariate analysis to evaluate the gradual changes in the neural representations of faces, from the coarse level of face and familiar face encoding to a more fine-grained level of representing familiar face subcategories and familiar face identities. Additionally, we examined whether the cortical areas involved in such representations show a specific topologic organization.

## Materials and Methods

### Subjects

Sixteen human adult subjects (11 males, 5 females, mean age = 26.29 ± 3.99 years) with normal vision were recruited for the imaging sessions. Informed written consent was obtained from each subject. All experimental procedures conformed to National Institutes of Health guidelines and were approved by the local ethics committee (approval number 99/60/1/1730).

In order to estimate the sample size, we ran a pilot experiment with nearly similar design to the main experiment. Collected data from this experiment was used for power analysis. In this analysis, the effect size calculation was based on the activation values in a PCC region (Brodmann area 23). α and β cutoff values were 0.05 and 0.2, respectively. The result indicated that 15 subjects were sufficient to detect the familiarity information.

### Visual stimuli

**Figure 1.**
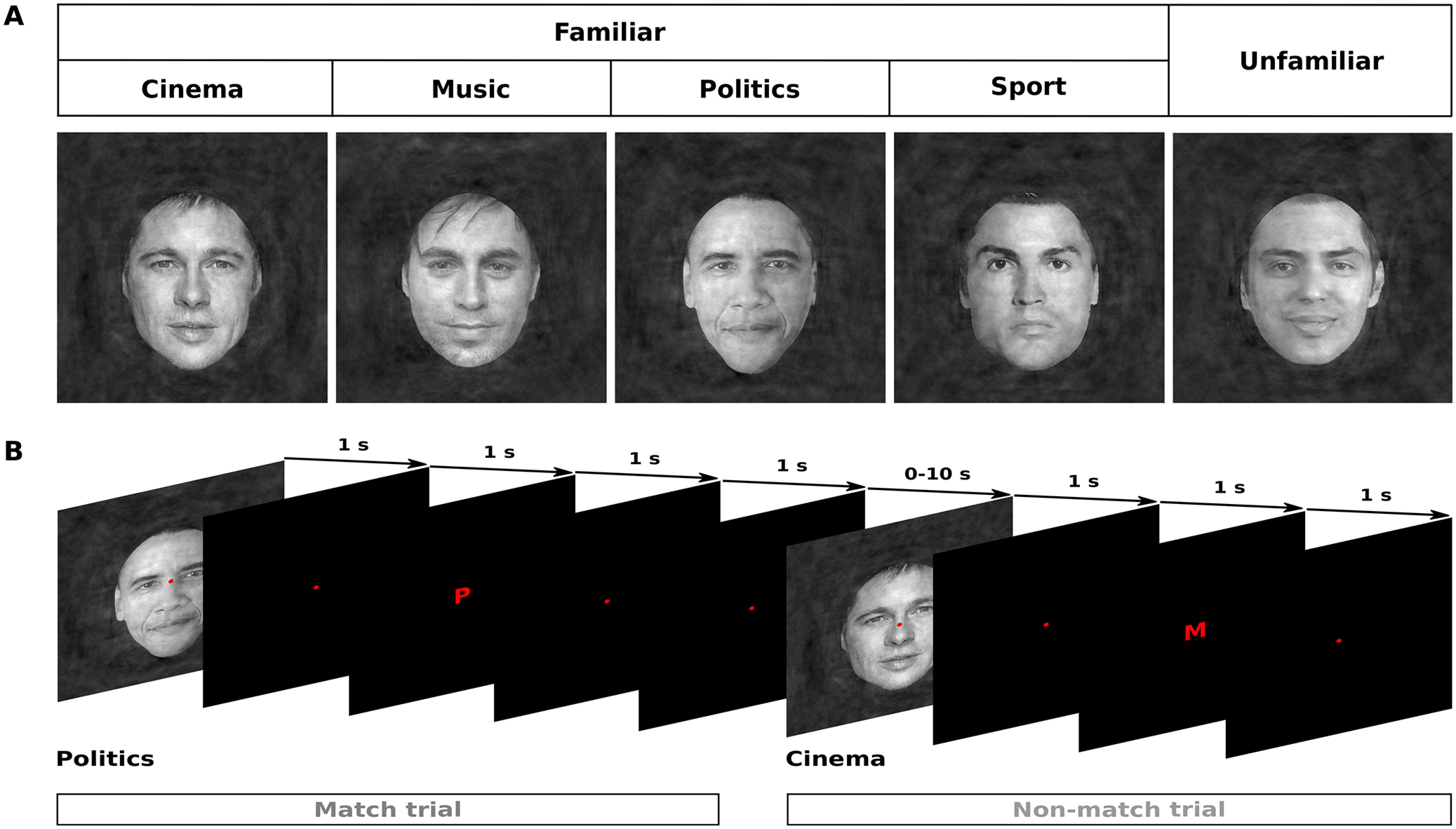
Face stimuli and task design. ***A***, Stimuli consisted of images from two main categories of faces: familiar faces and unfamiliar faces. The familiar face category included celebrity faces from four subcategories: cinema, music, politics, and sport. A red fixation point was positioned at the center of each image. ***B***, The sequence of events in a functional run. Each stimulus trial was composed of a face event (1-s face stimulus presentation, followed by a 1-s blank) and a letter event (1-s letter presentation, followed by a 1-s blank). The letter indicated a category label (e.g., P, politics; M, music, etc.). During the letter event, subjects had to compare the category label with the actual category of the face stimulus and report their match/non-match response by pressing a key on the response box. In addition to the stimulus trials, a number of null trials (delay periods) with variable durations were presented throughout the run.

**Figure 2.**
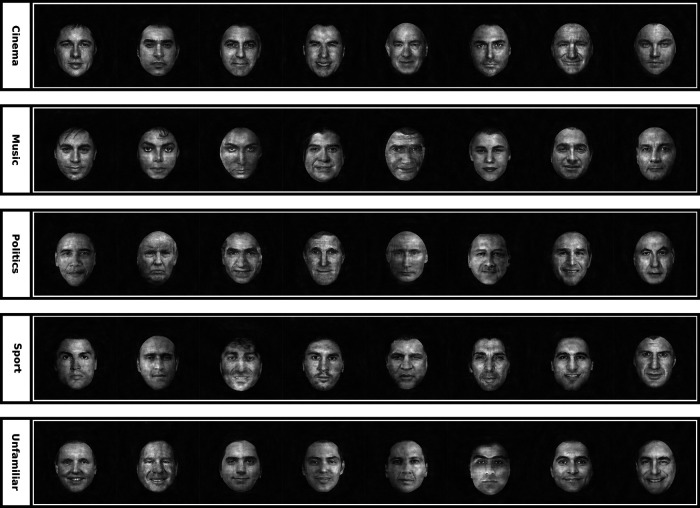
All samples of face stimuli used in the main fMRI experiment. The stimulus set contained faces of famous actors (cinema category), famous singers (music category), famous politicians (politics category), famous football players (sport category), and unfamiliar persons (unfamiliar category). There were eight images in each category. Images of all identities did not differ by any distinctive features such as gender, visual view, gaze direction, or facial expression. Also the images did not include characteristic features such as beard, mustache, or glasses. All images were transformed to grayscale format, masked with an oval-shaped aperture, and equalized with respect to low level visual features such as size, brightness, contrast, luminance histogram, and power spectrum.

Stimuli consisted of images from two main categories of faces: familiar faces and unfamiliar faces (Fig. 1*A*). The familiar face category included celebrity faces from four subcategories: cinema, music, politics, and sport. Familiar faces of each subcategory were selected based on high ratings of familiarity for our subjects (some of them were national celebrities). Unfamiliar faces were selected from celebrities that were unfamiliar for our subjects. In total, 40 different faces (4 × 8 familiar + 8 unfamiliar) were selected. All faces were male. Figure 2 shows all face images.

**Figure 3.**
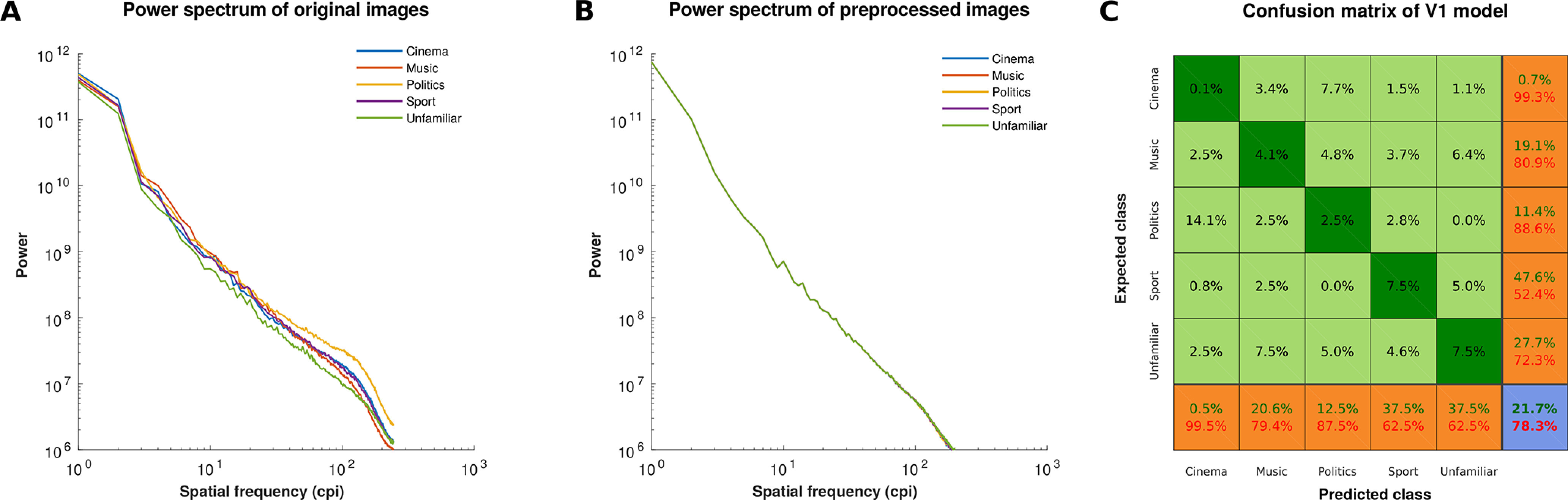
Spatial frequency matching of face stimuli, and results of the V1 model. ***A***, ***B***, The log-log plots show the averaged power of the Fourier transform at each spatial frequency for images of each face category before (***A***) and after (***B***) power spectrum equalization in the SHINE toolbox. The 1-D power spectra were obtained by averaging power across all orientations (i.e., radial averaging) in the 2-D power spectra of the images. ***C***, The confusion matrix summarizes the performance of V1 model in classifying the preprocessed face images into five semantic categories/classes. The rows of the matrix correspond to expected classes, and the columns of the matrix correspond to predicted classes. The diagonal cells show the pattern of correct classifications, and the off-diagonal cells show the pattern of misclassifications. The metrics of positive predictive value and FDR are shown in the last row, and the metrics of true positive rate and false negative rate are shown in the last column. The overall accuracy and error of the multiclass classification are shown in the blue cell (the values in green and red colors, respectively).

Original face images, which were obtained from the Internet, were frontal views of faces with direct gaze, natural hairstyle, and approximately neutral expression. Background of faces was segmented and filled by black color. Faces were then converted to the grayscale format, and their size was adjusted so that they can fit within an oval-shaped frame (the horizontal and vertical visual angles of the frame were 6.5° and 9°, respectively). The hairs were almost covered by this mask. The framed faces were embedded within a squared-shape black area (size = 12.5° visual angle), then the brightness, contrast, luminance histogram, and power spectrum of all face stimuli (including their surrounding black area) were physically matched/equalized using the Shine toolbox (Fig. 3*A*,*B*; Willenbockel et al., 2010). Finally, a red fixation point (radius = 1° visual angle) was superimposed at the center of face stimuli, and the stimuli were presented on a uniform black screen.

To quantitatively measure the discriminability of face categories based on low-level visual features (such as retinotopic shape information and spatial frequency), we evaluated the performance of a primary visual cortex (V1) model in classifying faces into five semantic categories. For this, we first simulated V1 cortical neurons using HMAX model (Serre et al., 2007). Images were modeled with a set of simple-like (S1) and complex-like (C1) cells in the second layer of the HMAX model, then the responses of these cells were used as input patterns for classification of images. Linear support vector machine (SVM), which was implemented in the LIBSVM toolbox, was used as a classifier for multiclass classification. The classification performance was assessed using leave-one-sample-out cross-validation method, in which one sample from each class was used for testing the classifier, and all remaining samples were used for training the classifier. The overall performance of the classifier was 21.7% correct rate, which was not significantly different from the chance-level performance (20%; *p* > 0.05, permutation test). Additionally, to investigate the contribution of each of pairwise comparisons to total performance, the confusion matrix was plotted (Fig. 3*C*). The correct rate for most pairwise comparisons was around the chance-level performance. Thus, the observed total accuracy was not dependent on a specific comparison. Overall, these analyses confirmed that the face categories were not distinguishable based on low-level pixel-wise information.

Faces in our stimulus set varied in terms of facial expressions. Using an online questionnaire, we asked 30 participants to indicate whether each face had a happy expression, a neutral expression, or an expression other than these two. A face was defined as a happy face if the percentage of “happy response” was above 60%. Similarly, a face was defined as a neutral face if the percentage of “neutral response” was above 60%. The effect of facial expression variables was removed during the analysis of functional data.

### Experimental procedure

Familiarity of each subject with face stimuli was quantitatively assessed through an online survey/questionnaire. Only subjects who were able to categorize all images of familiar faces were recruited in the study. In the scanning session, subjects completed 16 functional runs of the main fMRI experiment (run duration = 260 s) and two functional runs of the category localizer experiment (run duration = 216 s). Before scanning and outside the scanner, subjects were presented with all the 40 face stimuli (including familiar and unfamiliar faces), and they practiced the task of the main experiment.

A fast event-related fMRI design was used for presenting the stimuli in each functional run of the main experiment. An example functional run is shown in Figure 1*B*. The sequence of stimuli contained 40 stimulus trials (eight repetitions of five category conditions) and 35–39 null trials. Null trials were blank periods in which only fixation point was presented on a uniform black screen. To have the most optimized estimation of hemodynamic response, the order of stimulus and null trials for each run was generated using the FreeSurfer OptSeq2 algorithm (http://surfer.nmr.mgh.harvard.edu/optseq/). The first 10 and the last 4 s of the sequence were always nulls. Duration of stimulus trials was 4 s, and duration of null trials varied between 0 and 10 s. Each stimulus trial included a face event and a letter event, which were presented sequentially. In the face event, a randomly-chosen face stimulus (one out of 40 possible examples) was presented for 1 s, followed by a 1-s blank. In the letter event, a letter (size = 0.5° visual angle) was presented at the center of screen for 1 s, followed by a 1-s blank. The letter was either C, M, P, S, or U representing a category name. The reason for using the first letter of the category names (instead of the whole names) was to minimize confounding visual activations not related to face and semantic representations. For each stimulus category, half of the stimulus trials were “match trials” (the letter correctly represented the stimulus category), and the remaining stimulus trials were “non-match trials.” The order of match and non-match trials was random. Subjects were instructed to make a judgment about the category of each face stimulus and perform a match/non-match task. They indicated the match/non-match response by pressing a key with their right or left index finger. The responding hand for the match (and non-match) response was alternated and counterbalanced across subjects. Subjects were also asked to fixate on the fixation point throughout the scans. This design ensured that subjects paid attention to the semantic category of the face stimuli without making an association between a particular motor response and a particular face category. Such associations could impose problems during the multivariate pattern analysis (MVPA) of fMRI responses.

In the same fMRI session, a category localizer was used to localize face-selective regions including OFA, FFA, and ATFP. In each localizer run, four repetitions of three category blocks (faces, scenes, and objects) were presented. In each repetition, the order of blocks was random. Stimuli included grayscale images of faces (male and female faces), objects (e.g., tools, cars, and chairs) and scenes (indoor and outdoor scenes). In each block, 20 images were presented sequentially. Each image lasted 750 ms, followed by a blank interval of 50 ms. Subjects were instructed to report any spatial wiggle (a small jitter) in images. There was an 8-s fixation block in the beginning, middle, and end of each run.

### Data acquisition

Data were collected using a Siemens MAGNETOM Prisma 3T scanner with a 64-channel head coil at the National Brain Mapping Laboratory (NBML; Tehran, Iran). Subjects laid back in the scanner and viewed back-projected screen via a mounted mirror over the head coil. All stimuli were presented using MATLAB and Psychtoolbox (http://psychtoolbox.org/).

For each subject, a whole-brain anatomic scan was acquired using T1-weighted MP-RAGE sequence (TR = 2 s, TE = 3.47 ms, isotropic voxel size = 1 × 1 × 1 mm^3^, 256 sagittal slices, flip angle = 7^0^, GRAPPA acquisition with acceleration factor = 2).

The functional scans for the main experiment and category localizer were based on a GE-EPI sequence (TR = 2 s, TE = 30 ms, voxel size = 3.5 × 3.5 × 3.5 mm^3^, 34 semi-axial slices, distance factor = 10%, flip angle = 90^0^, GRAPPA acquisition with acceleration factor = 2). The slices were obtained in an even-odd interleaved order. The first three volumes of each run were discarded to allow for MR signal equilibration.

### Data analysis

Using recon-all in FreeSurfer (http://surfer.nmr.mgh.harvard.edu/), structural T1 images of each subject were processed, subcortical structures were automatically segmented, and cortical surfaces were computationally reconstructed. FsFast (https://surfer.nmr.mgh.harvard.edu/fswiki/FsFast) was used for preprocessing and voxel-wise analysis of functional data.

#### Preprocessing

Functional data were first skull-stripped using FSL's brain extraction tool to generate a brain mask (Smith, 2002). Then using the middle time point of each run as the reference and applying AFNI's motion correction algorithm, the functional images were aligned (Cox, 1996). For each subject, only runs with <1 mm motion in any direction were included in the fMRI analyses. Using this criteria, one run was excluded in three subjects. In the next step, intensity values of all voxels inside the brain mask were converted to a standard intensity scale. For this, the mean intensity of all voxels across all time-points was first computed. Then, the intensity value of each voxel at each time point was divided by the mean intensity and multiplied by 100. The functional volumes were then rigidly co-registered to the same-subject anatomic volumes using boundary-based registration method.

#### Univariate analysis

In the univariate analysis, the preprocessed functional data were resampled/projected to an average cortical surface (“fsaverage”) using spherical transformation. Projected data were then spatially smoothed using a Gaussian filter [full width at half maximum (FWHM) = 6 mm]. For each surface vertex, activations for different category conditions were calculated using a general linear model (GLM). In this model, time-series of all runs within a session were concatenated, and a design matrix composed of task regressors and run-related nuisance regressors was constructed. Time-series were whitened by removing temporal autocorrelations. Task regressors represented temporal patterns of five face events (cinema, music, politics, sport, and unfamiliar faces) and one letter event, which were all convolved with a canonical hemodynamic response function. The head motion parameters produced during realignment were used in the GLM as nuisance regressors to account for residual effects of subjects' movements. Additional nuisance variables included linear trends, quadratic trends, and mean confound. We also ran a version of GLM in which the facial expression variables were regressed out. Before estimating β values of the model, the first four time-points of each run were discarded to avoid inhomogeneity effects of the magnetic field. Finally, β values for the task regressors were obtained.

We performed a two-stage surface-based univariate analysis to localize regions selective for familiar faces in the common anatomic space (fsaverage). In the subject-level analysis, statistical maps were computed by *t* test comparison between β values in the contrast of familiar versus unfamiliar faces. Then, in the group analysis, the familiar face regions were defined based on mixed-effects averaging of individual subject maps. The group-average map was thresholded at false discovery rate (FDR)-corrected *p* < 0.05.

#### Multivariate analysis

In the MVPAs, to prevent loss of fine-grained pattern information, preprocessed data were analyzed in the subjects' native anatomic space without applying any spatial smoothing. Using a procedure described in the univariate analysis, β values associated with task events were estimated. Here, estimation of β values was performed in the voxel space and separately for each run. To evaluate pattern information for subcategories of familiar faces, the design matrix included 6 distinct task regressors for five face events (cinema, music, politics, sport, and unfamiliar faces) and one letter event. To evaluate pattern information for familiar face identities, the design matrix included 41 distinct task regressors for 40 individual face events and one letter event. After fitting the GLM, estimated β values of all voxels within a given region of interest (ROI) mask were concatenated to form an fMRI pattern vector for each condition in each run. To remove the common response pattern, β values were normalized by subtracting the mean across conditions. To test whether neural patterns could distinguish between different stimulus conditions, we used two decoding approaches: correlation method and SVM classification method.

In the correlation method, 16 runs of each subject were split into two complementary sets of 8 runs (e.g., odd and even runs). Within each set and for each condition, fMRI activation patterns were averaged across runs. In each ROI mask, patterns of activities (one pattern for each condition) in one half of data were correlated with patterns of activities in the other half of data, which resulted in a split-data representational similarity matrix (sdRSM) with the size N×N (rows and columns of the matrix corresponded to *N* conditions). Pearson's *r* was used for computing pairwise correlations. To avoid arbitrariness in data splitting, the sdRSMs were calculated for 100 permutations of run splitting, which were selected randomly out of all possible permutations. The correlation values were then averaged across all permutations. The amount of categorical information in each ROI was quantified using a category discriminability index (CDI). The CDI metric was defined as the average of within-condition pattern correlations (diagonal elements of sdRSMs) minus the average of between-condition pattern correlations (off-diagonal elements of sdRSMs).

To evaluate different hypotheses about neural representations, categorical models were designed, and sdRSMs were correlated with these model matrices using Kendall's tau rank correlation (Khaligh-Razavi and Kriegeskorte, 2014; Nili et al., 2014, 2020). To examine whether the correlations between categorical models and sdRSMs were significant, estimates of all subjects were combined to perform random-effects group-level statistical analysis using *t* test. To correct for multiple comparisons, the Benjamini–Hochberg procedure for controlling the FDR (FDR correction) was used (Benjamini and Hochberg, 1995).

In the classification method, the functional runs were split into two independent sets. In each round of data splitting, the training set included 15 runs, and the test set included the left-out run. The two sets of runs had independent stimulus sequences and independent preprocessing, the two important factors for avoiding circular inferences and overfitting. To test whether categorical information could be encoded in each ROI mask, six pairwise classifiers (cinema vs music, cinema vs politics, and so on) were trained on responses to four category conditions (cinema, music, politics, and sport categories) in the training set. For training the classifiers, SVM with a linear kernel was implemented in LIBSVM. SVM has several advantages over other classifiers; it does not depend on the distribution of training data, and it can handle limited data in high-dimensional spaces (Allefeld and Haynes, 2015). Decision boundaries obtained from training the classifiers were applied to an independent test set (a single run), so that the generalizability of the decision boundary could be evaluated. The classification performance was based on leave-one-run-out cross-validation method, and it was defined as an average performance across all folds of cross-validation. Finally, to determine whether significant information was encoded for different stimulus classes, accuracy of each classifier was compared with the chance-level accuracy (50% performance) using *t* test across subjects.

#### Searchlight analysis

A surface-based searchlight method was used to localize regions that could distinguish between activity patterns of different stimulus conditions. This analysis was performed in the native space of subjects using the CosmoMVPA toolbox (Oosterhof et al., 2016). Specifically, a curved cylinder was defined around each point/vertex between two cortical surfaces (FreeSurfer's “pial” and “white” surfaces), which sampled a constant number of voxels (100 voxels in our analysis). Then, the estimated β values of these voxels were concatenated to generate the response patterns for different conditions. Pattern analysis was performed for all possible regions on the cortical surface using “correlation with the categorical model” approach. For each region, the correlation value was assigned to the central vertex of that region. Finally, a vertex-wise map of correlation values (a map of categorical information) was obtained in each subject, and the results were transformed into the standard space (fsaverage) for group analysis.

#### ROI definitions

**Figure 4.**
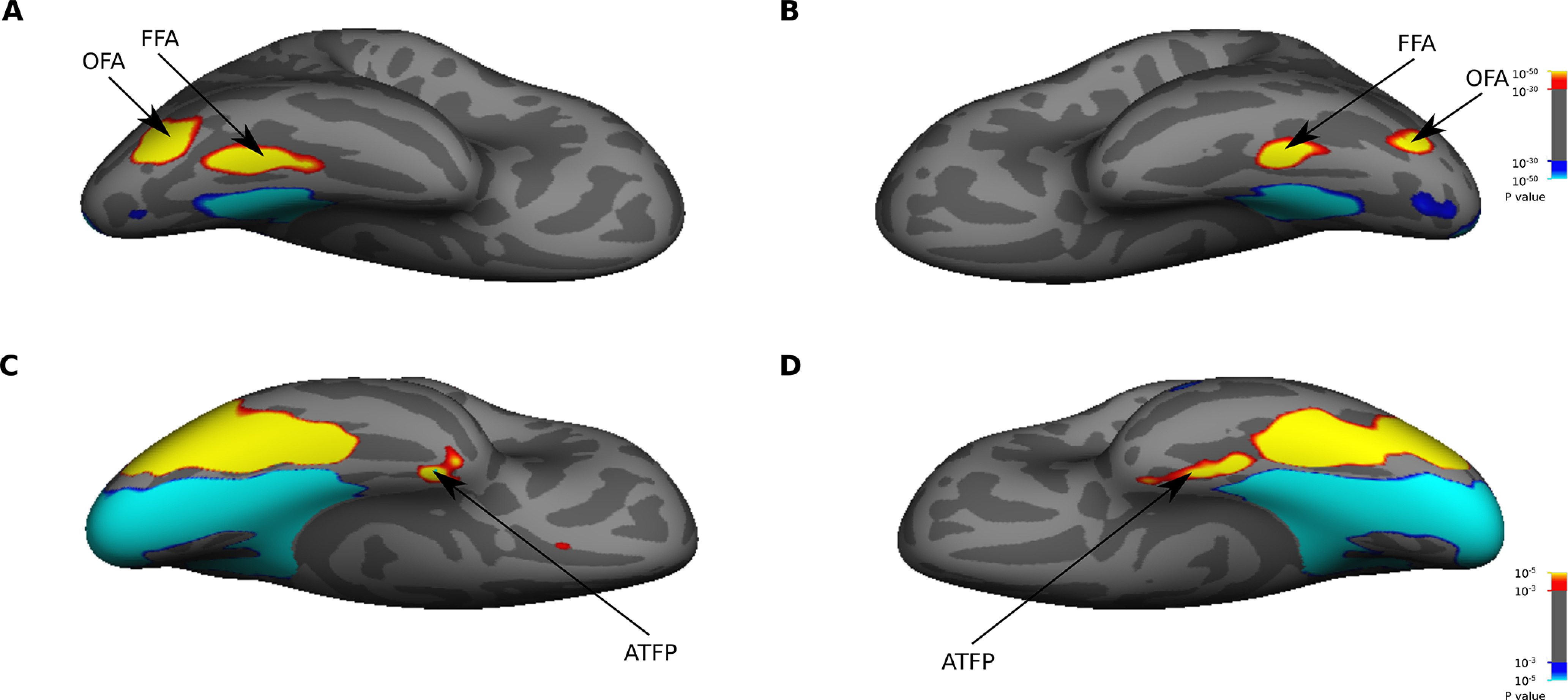
Face-selective ROIs. The maps show significant activations for face (red/yellow) versus scene (blue/cyan) stimuli in right (***A***, ***C***) and left (***B***, ***D***) hemispheres. The activation maps are based on mixed-effects group-average of face localizer data from 16 subjects, and are displayed on ventral views of an inflated cortical surface (the fsaverage surface). The colorbars indicate the significance values. For thresholding the significance maps, two values were used: a high threshold for defining the strongly activated face-selective areas OFA and FFA (***A***, ***B***), and a low threshold for defining the weakly activated face-selective area ATFP (***C***, ***D***). These ROIs were projected/transformed from the fsaverage space to the native anatomic space of each subject.

Classic face-selective areas, including OFA, FFA, and ATFP, were localized using category localizer scans. We performed the same steps of preprocessing and GLM analysis as the univariate analysis, except that the design matrix included distinct task regressors for three category blocks: faces, scenes, and objects. The face-selective ROIs were defined based on the contrast of faces versus scenes. For localizing OFA and FFA, the group-average maps were thresholded at *p* < 10^–30^ (Fig. 4). OFA/FFA were identified as cluster of voxels in posterior/anterior parts of the bilateral fusiform gyrus showing higher activation for faces than scenes. For localizing ATFP, the group-average maps were thresholded at *p* < 0.001 (Fig. 4). The activation in ATFP is generally weak presumably because of susceptibility-induced loss of MR signal in the ATL (Rajimehr et al., 2009). Thus, a low threshold of statistical significance was used for detecting ATFP. The anatomic region of Brodmann area 23 (one of the subdivisions of PCC) was also defined using PALS-B12 atlas of human cerebral cortex (Van Essen, 2005) on the fsaverage surface. For the multivariate analysis, these surface-based ROIs were projected from the common anatomic space onto native anatomic space of each subject.

In a control analysis, discriminability of face categories was assessed in the low-level visual cortical area V1. The visually responsive V1 ROI was defined in each hemisphere of each subject using the following procedure. First, the maps of visually responsive voxels were derived based on the contrast of faces versus fixation (faces > fixation) in the main fMRI experiment. Then, the resulting maps were intersected with anatomic V1 ROIs generated by the FreeSurfer parcellation (Hinds et al., 2008). Two subcortical ROIs (hippocampus and amygdala), which were obtained for each subject through FreeSurfer's automated subcortical segmentation, were also included in the multivariate ROI analysis.

#### Face-selective maps from Human Connectome Project (HCP) database

To compare our activations with a broader network of face areas, face-selective maps were obtained from the HCP task fMRI dataset (https://www.humanconnectome.org/study/hcp-young-adult). Details of data acquisition have been described elsewhere (Glasser et al., 2016). We used data from the working memory task. For the working memory task, 787 subjects underwent two runs of functional scanning while performing an N-back (0–2 back) task. In each run, subjects were presented with blocks of images from four categories (faces, places, tools, and body parts). The blocks had either 0-back or 2-back working memory task, as indicated by a cue at the beginning of the block. Each block contained 10 trials. In each trial, an image was presented for 2 s, followed by a 500-ms intertrial interval. Data were preprocessed and analyzed using the HCP pipelines (Glasser et al., 2013). Specifically, the preprocessed data were projected to a standard space, the surface data were spatially smoothed by a 2-mm FWHM Gaussian kernel, and GLM was used to estimate functional activities in each vertex. In the subject-level analysis, face-selective maps were obtained based on the contrast of faces versus all other categories, collapsing across the memory conditions. Maps of individual subjects were averaged in the standard space. The face-selective vertices were defined as the top 1% of vertices, which had the highest z values in the contrast of faces versus all other categories (Abbasi et al., 2020). The 99th percentile corresponded to the cutoff-point z value of 12.38 in the group-average map. The final map was transformed into the fsaverage surface.

#### Topographic relationship between activation patterns

To quantify the topographic relationship between representations for familiar faces, familiar face subcategories, and familiar face identities, we repeated the univariate and multivariate searchlight analyses for 500 permutations of run splitting, which were selected randomly out of all possible permutations. The analyses were restricted to a predefined mask in medial occipito-parietal cortex. The mask included three anatomic ROIs (isthmus cingulate cortex, precuneus, and PCC) from FreeSurfer's Desikan–Killiany atlas (Desikan et al., 2006). For familiar faces, the peak activations (vertices with the highest values) were derived from the two-step univariate analysis on 500 sets of runs, each containing half of the data. To obtain these sets, 250 random splitting of runs was performed, and two complementary sets from each splitting were used. For familiar face subcategories and familiar face identities, the peak activations were obtained using the correlation method described in the multivariate analysis. The correlation values were calculated for each subject and each permutation, then they were averaged across subjects. In each permutation, the vertex with the highest value was selected. This procedure provided three distributions of points (500 points for each type of face representation).

To statistically investigate the separation between distributions of peak activations, we first extracted each peak's spatial information within the White surface right-anterior-superior (RAS) coordinate system, then differences between group medians along three coordinate axes were examined using multivariate Kruskal–Wallis test. Pairwise comparisons were also performed using non-parametric two-tailed Mann–Whitney *U* test. To correct for multiple comparisons, the Bonferroni correction method was used. In another approach, a dissimilarity matrix (1500 × 1500) was constructed based on the Euclidean distances between the peaks, then an analysis of similarity (ANOSIM; Clarke, 1993) was performed using the vegan package in R. For *post hoc* tests, pairwise ANOSIMs between all pairs of groups were conducted and *p* values were corrected for multiple comparisons using the Bonferroni correction method. ANOSIM is a non-parametric test, which statistically compares similarities of two or more groups based on a distance measure. It provides an R statistic which represents the difference between the mean of ranked dissimilarities between groups and the mean of ranked dissimilarities within groups. To statistically evaluate the significance of R, a permutation test was performed (number of permutations = 1000).

#### Functional connectivity analysis

In the functional connectivity analysis, we aimed to investigate the whole-brain patterns of functional connections (functional couplings) for the semantic face areas of left PCC. We used a seed-based approach in which four seed regions were defined as surface-based circular regions (with radius of ∼5 mm) around the peak coordinates/vertices derived from univariate and MVPA analyses. For vertices within the seed regions and all other vertices of cortex, the fMRI time-series were obtained using the following procedure. The functional data of each subject were preprocessed using standard preprocessing steps (for further details, see above, Preprocessing), resampled to an average cortical surface (“fsaverage”), and spatially smoothed using a Gaussian filter (6-mm FWHM). The preprocessed data were then entered a GLM model. In the GLM model, a stimulus-related variable was regressed out. Additionally, head motion parameters and signals from white matter (WM) and ventricular CSF (VCSF) were regressed out as nuisance variables. For the signals of WM and VCSF, we used top five principal components estimated by PCA on the time-series of voxels in these structures. The GLM residuals were used as time-series in the functional connectivity analysis. This approach would be qualitatively equivalent to using stimulus-free resting-state fMRI data (Fair et al., 2007). For each vertex, the time-series of all functional runs were concatenated after transforming to z scores. For each seed region, the time-series were averaged across vertices within the region.

The functional connectivity maps were obtained by computing the Pearson correlation between mean time-series of each seed region and the time-series of all vertices in cortex. The Fisher's r-to-z transform {z = 0.5 × *Ln*[(1 + r)/(1 − r)]} was applied to the correlation values, then the connectivity maps were averaged across subjects. The maps were thresholded at *r* = 0.12, which approximately corresponded to an average correlation value across all vertices in four maps, and the resulting masks (strongly connected vertices) were saved. To determine regions that were preferentially coupled to one seed region, a winner-take-all procedure was used (Silson et al., 2019b). In this procedure, the amount of functional coupling between time-series of seed regions and time-series of vertices in the masked area was estimated in each subject using a multiple regression method. Then, the estimated β values were averaged across subjects, and each vertex was assigned to a seed region which had the greatest β value. To statistically evaluate the differences in functional couplings between seed regions and face-selective areas in ventral temporal cortex, we conducted one-way repeated measures ANOVAs with seed region as a within-subject factor.

## Results

### Behavioral results

During the functional runs of the main fMRI experiment, subjects performed a match/non-match task. The overall performance and reaction time of subjects in this task were 96.31 ± 4.23% and 558 ± 54 ms, respectively. This high performance, which was observed in all individual subjects, confirmed that subjects were performing the task correctly. In the analysis of behavioral data, we found no significant difference in performance between the five category conditions [one-way ANOVA; *F*_(4,75)_ = 1.01, *p* > 0.05]. Likewise, there was no significant difference in reaction time between the five category conditions [one-way ANOVA; *F*_(4,75)_ = 0.31, *p* > 0.05]. These results indicated that the five face categories were matched in terms of task difficulty and attentional engagement of subjects.

### Cortical representations of familiar faces

**Figure 5.**
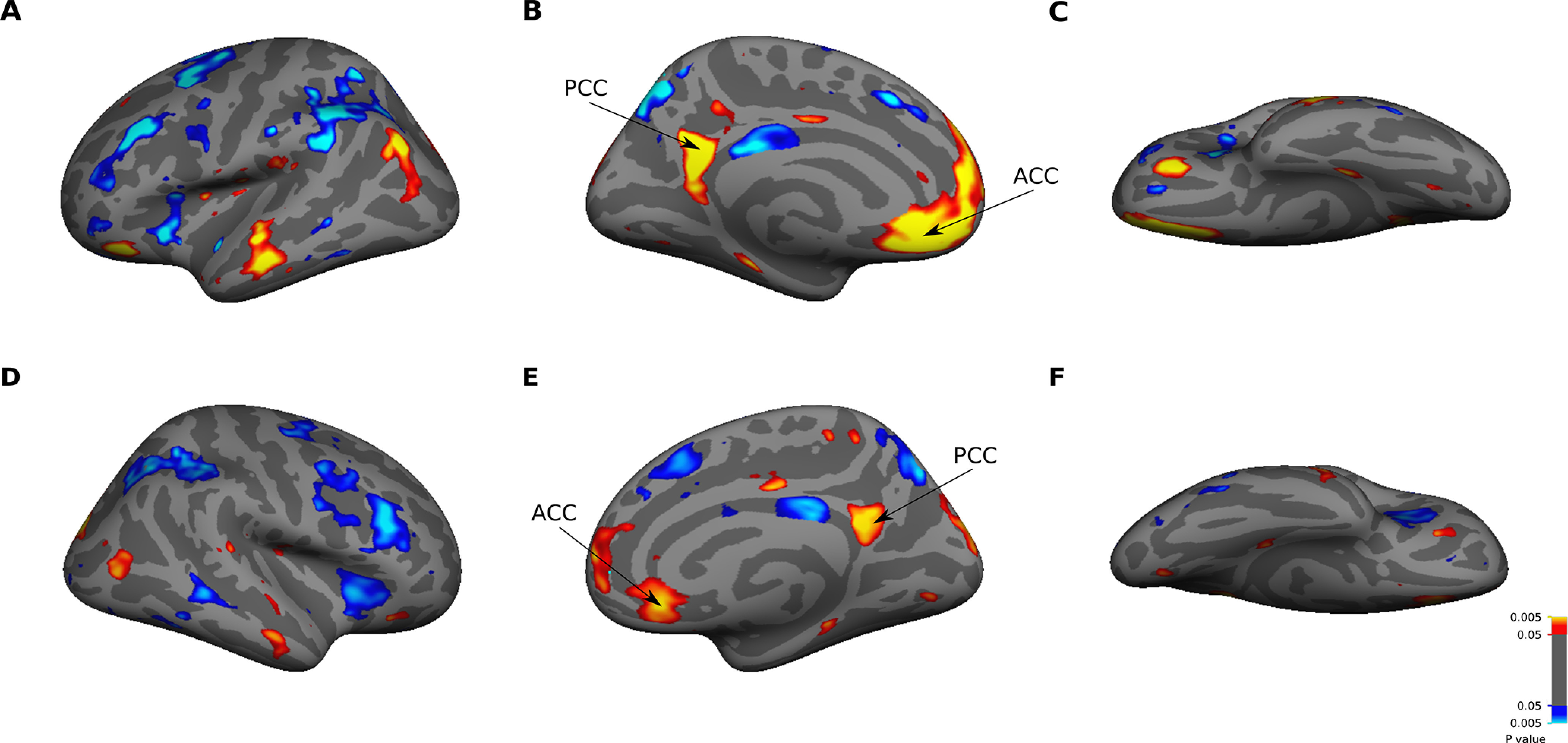
Whole-brain univariate analysis. The maps show significant (*p* < 0.05, *t* test, FDR-corrected) activations for familiar (red/yellow) versus unfamiliar (blue/cyan) faces in left (***A–C***) and right (***D–F***) hemispheres. The activation maps are based on mixed-effects group-average of data from 16 subjects and are displayed on lateral (***A***, ***D***), medial (***B***, ***E***), and ventral (***C***, ***F***) views of an inflated cortical surface (fsaverage surface). The familiarity activated two distinct cortical regions (PCC and ACC) in medial cortex of both hemispheres.

**Figure 6.**
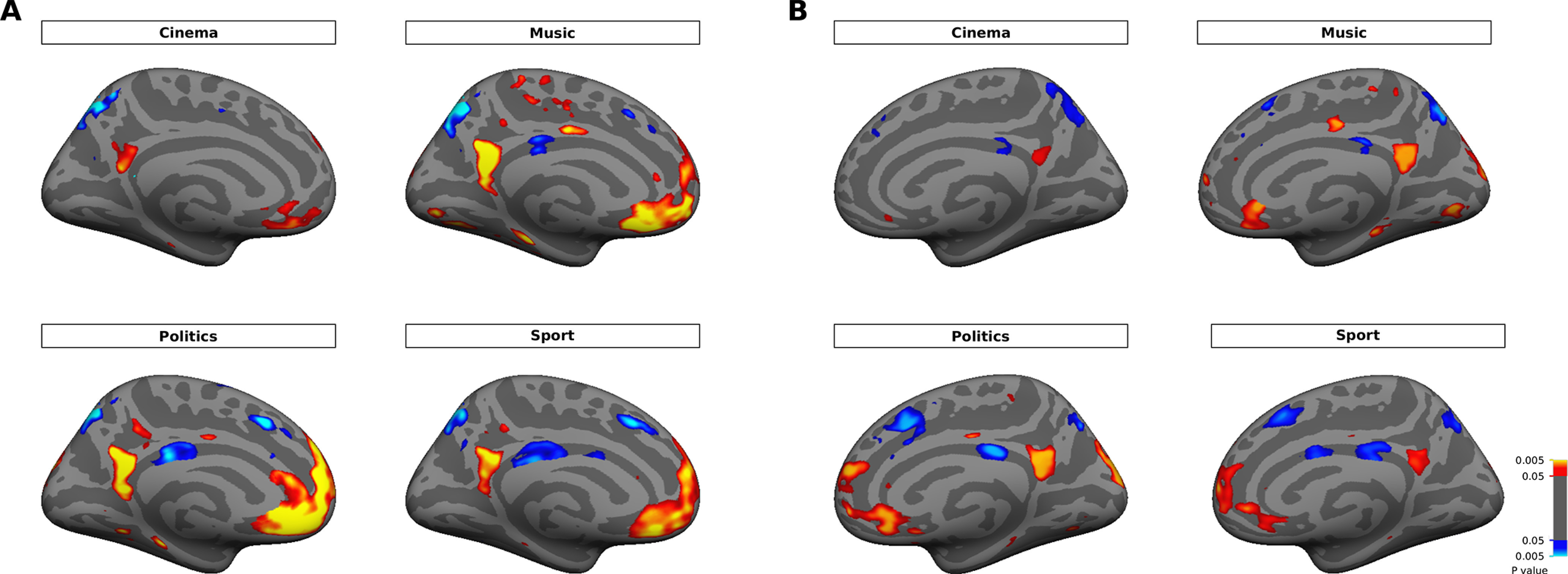
Univariate comparison between activities of familiar face subcategories versus unfamiliar faces. The maps show significant (*p* < 0.05, *t* test, FDR-corrected) activations for each subcategory of familiar faces (red/yellow) versus unfamiliar faces (blue/cyan). The group-average activation maps are displayed on medial views of the inflated cortical surface in left (***A***) and right (***B***) hemispheres.

Based on the univariate analysis, we defined cortical regions that were activated by familiar faces. In the group-average maps (Fig. 5), we found significantly greater activation in PCC and ACC for familiar than unfamiliar faces. The ACC activation extended to anterior medial prefrontal cortex (PFC) in the left hemisphere. PCC and ACC were consistently activated when we compared each subcategory of familiar faces versus unfamiliar faces (Fig. 6). Two other spots in the lateral part of ATL and temporo-parietal junction (TPJ) also showed modulation of activity by familiarity. PCC, ACC, lateral ATL, and TPJ were all located within the default mode network (DMN; Yeo et al., 2011). For all these regions, the selectivity to familiar faces was significant in both hemispheres. However, the extent of activity was more pronounced in the left hemisphere. On the ventral surface, face-selective areas OFA, FFA, and ATFP did not show a significantly higher response to either familiar or unfamiliar faces. The responses to familiar and unfamiliar faces were not significantly different from each other in early visual cortex, confirming that these faces were well matched in terms of low-level visual features.

### Cortical representations of familiar face subcategories

**Figure 7.**
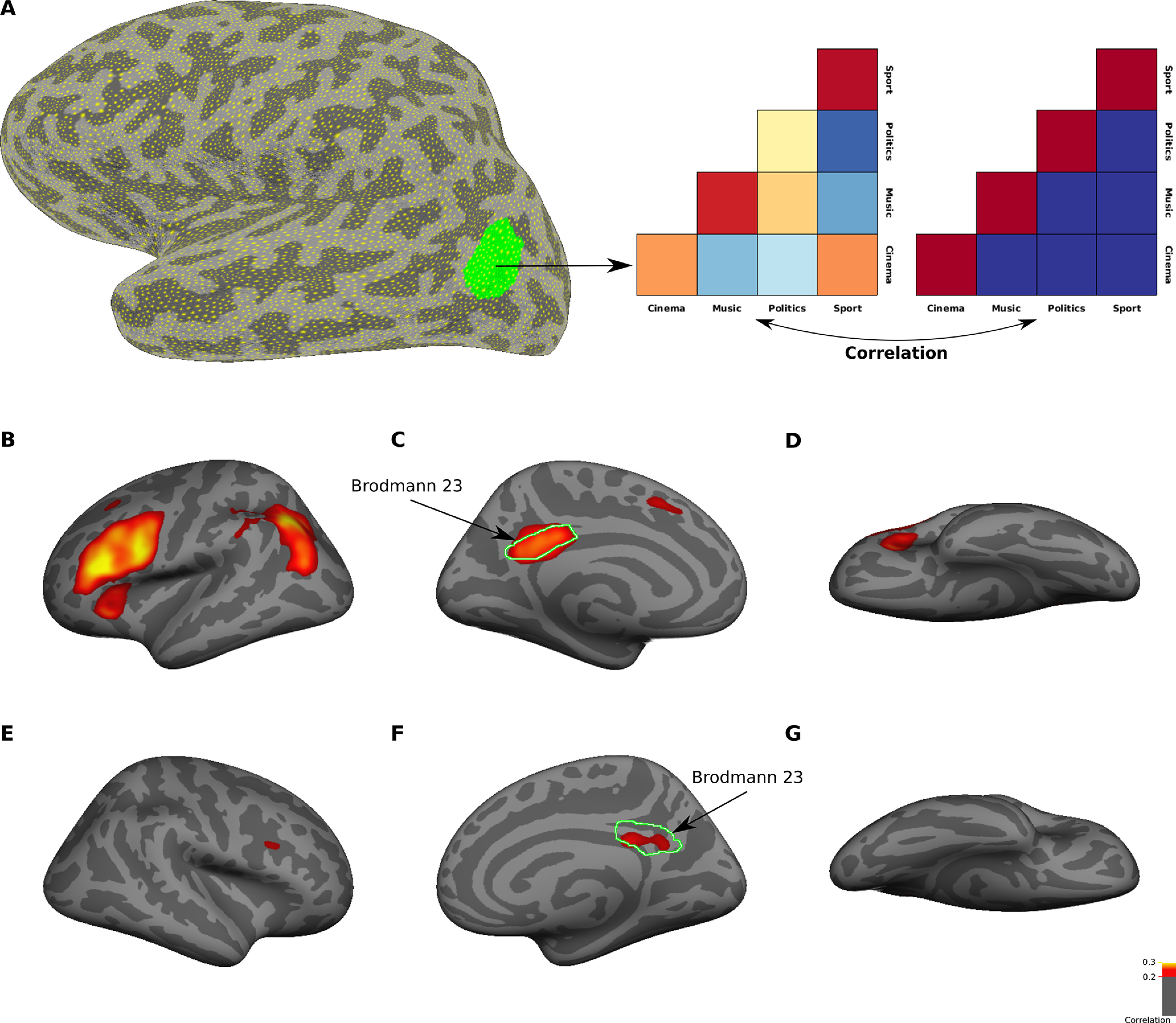
Whole-brain searchlight analysis for identifying regions that encode familiar face subcategories. ***A***, The procedure for surface-based searchlight analysis. For each surface mask, Kendall's tau correlation between neural sdRSM and categorical model was computed. Here, we used a model to test pattern information for familiar face subcategories. The green region is an example surface mask in a representative subject. ***B–G***, The maps show averaged correlation coefficient values from 16 subjects in left (***B–D***) and right (***E–G***) hemispheres. Only vertices with a significant (*p* < 0.05, *t* test, FDR-corrected) correlation are included in the maps. The correlation maps are displayed on lateral (***B***, ***E***), medial (***C***, ***F***), and ventral (***D***, ***G***) views of an inflated cortical surface (fsaverage surface). The border of Brodmann area 23 was defined in both hemispheres of fsaverage surface based on PALS-B12 atlas.

To identify regions which could distinguish between subcategories of familiar faces, we performed a whole-brain MVPA using a surface-based searchlight method (Fig. 7*A*). Specifically, we used “correlation with the categorical model” approach. For each region on the cortical surface, sdRSM with the size 4 × 4 was computed. Rows and columns of the matrix corresponded to four subcategories of familiar faces (cinema, music, politics, and sport). Each element of the matrix represented the correlation between response patterns for two category conditions in two complimentary sets of runs (e.g., cinema condition in odd runs and politics condition in even runs). An ideal categorical model was an identity matrix in which the diagonal elements had maximum correlation of one and the off-diagonal elements had zero correlation. The rank correlation between neural sdRSM and the categorical model indicated how much each region contained information about subcategories of familiar faces. The searchlight maps from group analysis are shown on the inflated surfaces of both hemispheres in Figure 7*B–G*. Subcategories of familiar faces could be decoded significantly in PCC of both hemispheres, in a region that highly overlapped with Brodmann area 23. Two other significant clusters were located in posterior parietal cortex (PPC) and lateral PFC (LPFC) of left hemisphere. On the ventral surface, no significant cluster was observed. The searchlight maps did not change after regressing out the facial expression variables (data not shown).

### ROI analysis

The results of searchlight analysis showed that categorical information of familiar face subcategories was strongly represented in Brodmann area 23, a subdivision of PCC. However, this analysis did not reveal any representations of categorical information in visual cortical regions. We therefore used an ROI analysis approach to further explore the sensitivity of visual cortical regions (including V1, OFA, FFA and, ATFP) to the categorical information of familiar faces. We also included Brodmann area 23 in the ROI analysis. The ROI analysis differs from the searchlight analysis in two important ways. First, the pattern analysis is performed for voxels within functionally (or anatomically) defined areas. These areas may not necessarily have a circular shape, as it is assumed in the searchlight analysis. Second, the number of voxels in a given ROI can be higher than a predefined number used in the searchlight analysis.

**Figure 8.**
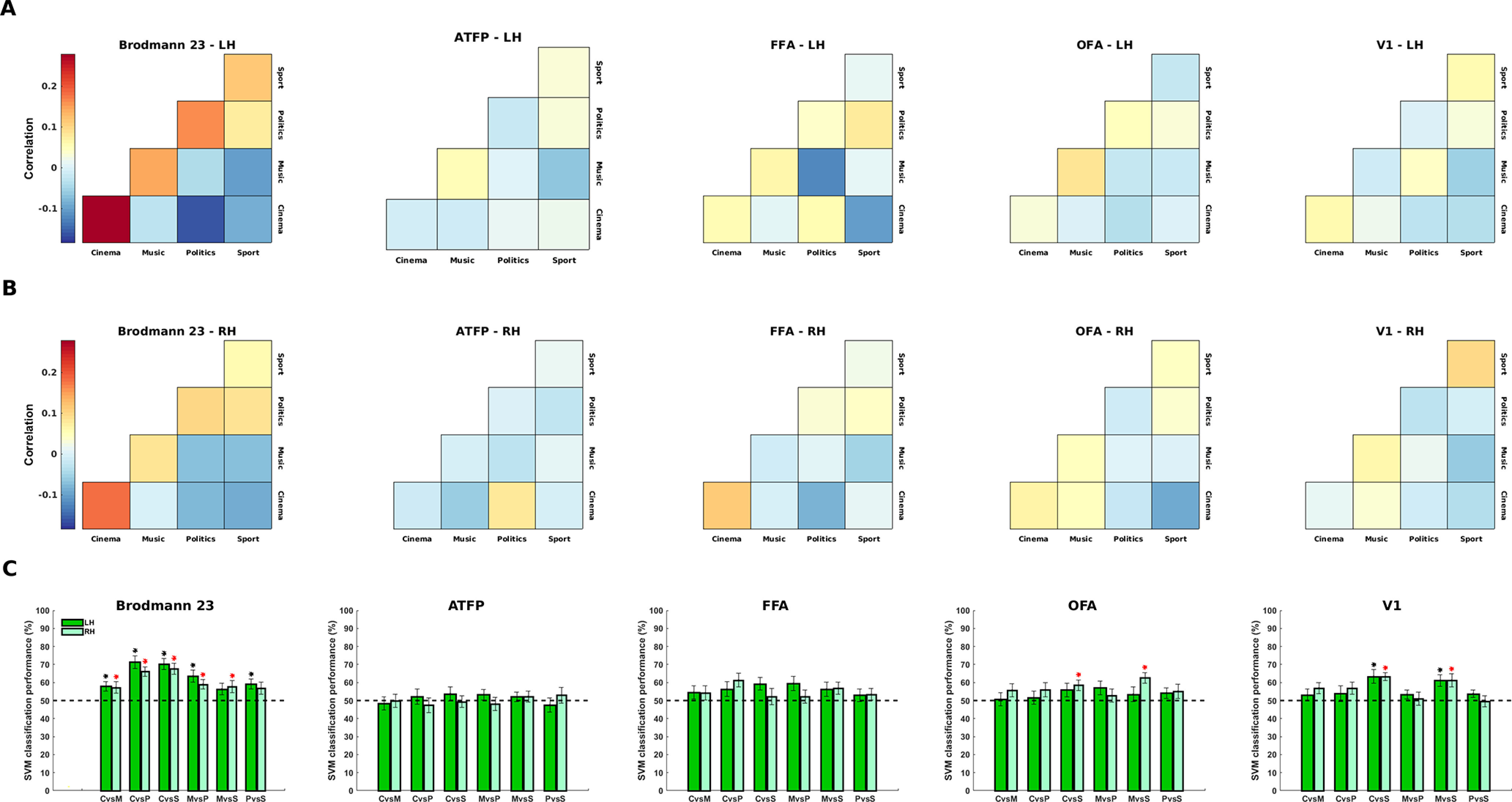
ROI-based MVPA for familiar face subcategories. From left to right, graphs depict results of multivariate analysis for different ROIs including Brodmann area 23, ATFP, FFA, OFA, and V1. The matrices in panels ***A***, ***B*** are averaged neural sdRSMs across 16 subjects in left (***A***) and right (***B***) hemispheres. sdRSM in each subject was obtained by averaging sdRSMs across 100 random permutations of run splitting. The matrices are triangular because corresponding elements in upper triangular and lower triangular parts of sdRSMs have been averaged. The color scale bar represents Pearson correlation coefficient values. Some of the correlation values are negative mainly because normalized data have been used (see Materials and Methods). The bar plots in panel ***C*** show SVM classification performance for six pairwise comparisons between face categories (C, cinema; M, music; P, politics; S, sport). Dark green and light green bars show results of classification analysis in left and right hemispheres, respectively. Black and red asterisks indicate significant performance (*p* < 0.05, *t* test, FDR-corrected) for dark green and light green bars, respectively. Dashed black line indicates the chance level of 50%. Error bars denote the SEM calculated across subjects.

Pattern information in each ROI was assessed using two different methods of MVPA: correlation method and classification method (see Materials and Methods). Figure 8 shows the results of ROI analysis. In the correlation matrices of Brodmann area 23, the activations were more similar in within-category conditions than between-category conditions (diagonal vs off-diagonal cells in the correlation matrix), especially in the left hemisphere. Such pattern was not observed in the correlation matrices of other ROIs. This result confirmed that Brodmann area 23, but not the selected visual cortical regions, contained information about familiar face categories. In Brodmann area 23 of both hemispheres, the SVM classification performance was also significantly higher than chance level in five (out of six) pairwise comparisons between face categories – again demonstrating that this area had separable representations of familiar face categories. In contrast, no pairwise comparisons showed significant performance in FFA and ATFP. Categorical information was weakly represented in OFA and V1. The SVM classification performance was significantly above chance level in two pairwise comparisons in right OFA, and in two pairwise comparisons in left and right V1. This result confirmed that low-level visual features, which were predominantly represented in V1, had minimal contributions to the discrimination of familiar face categories.

**Figure 9.**
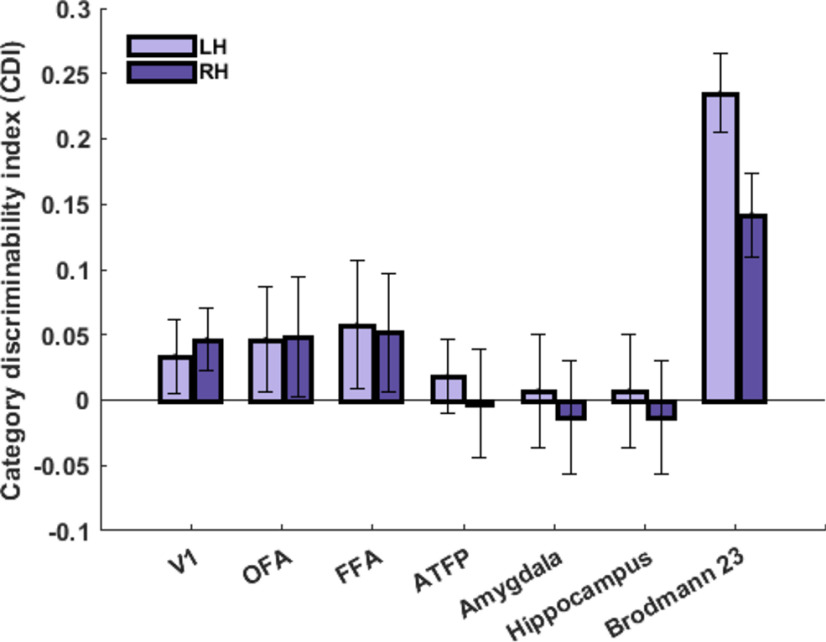
CDI for familiar face subcategories in selected ROIs. The bar plot shows CDI (a metric for encoding categorical information of familiar faces) in V1, OFA, FFA, ATFP, amygdala, hippocampus, and Brodmann area 23. Light purple and dark purple bars show CDI in left and right hemispheres, respectively. CDI was significantly greater than zero (*p* < 0.05, *t* test, FDR-corrected) only in Brodmann area 23. Error bars denote the SEM calculated across subjects.

In each ROI, the categorical information for familiar faces was also quantified using CDI metric (difference between the correlation values in diagonal and off-diagonal cells of the correlation matrix). In this analysis, we included two subcortical areas (amygdala and hippocampus), which were previously reported to have selective activation for familiar faces (Viskontas et al., 2009; Ramon et al., 2015). The result of this analysis is shown in Figure 9. As expected, Brodmann area 23 contained the highest amount of categorical information in both hemispheres. All other cortical areas (V1, OFA, FFA, and ATFP) and subcortical areas (amygala and hippocampus) contained weak and non-significant categorical information.

### Cortical representations of familiar face identities

**Figure 10.**
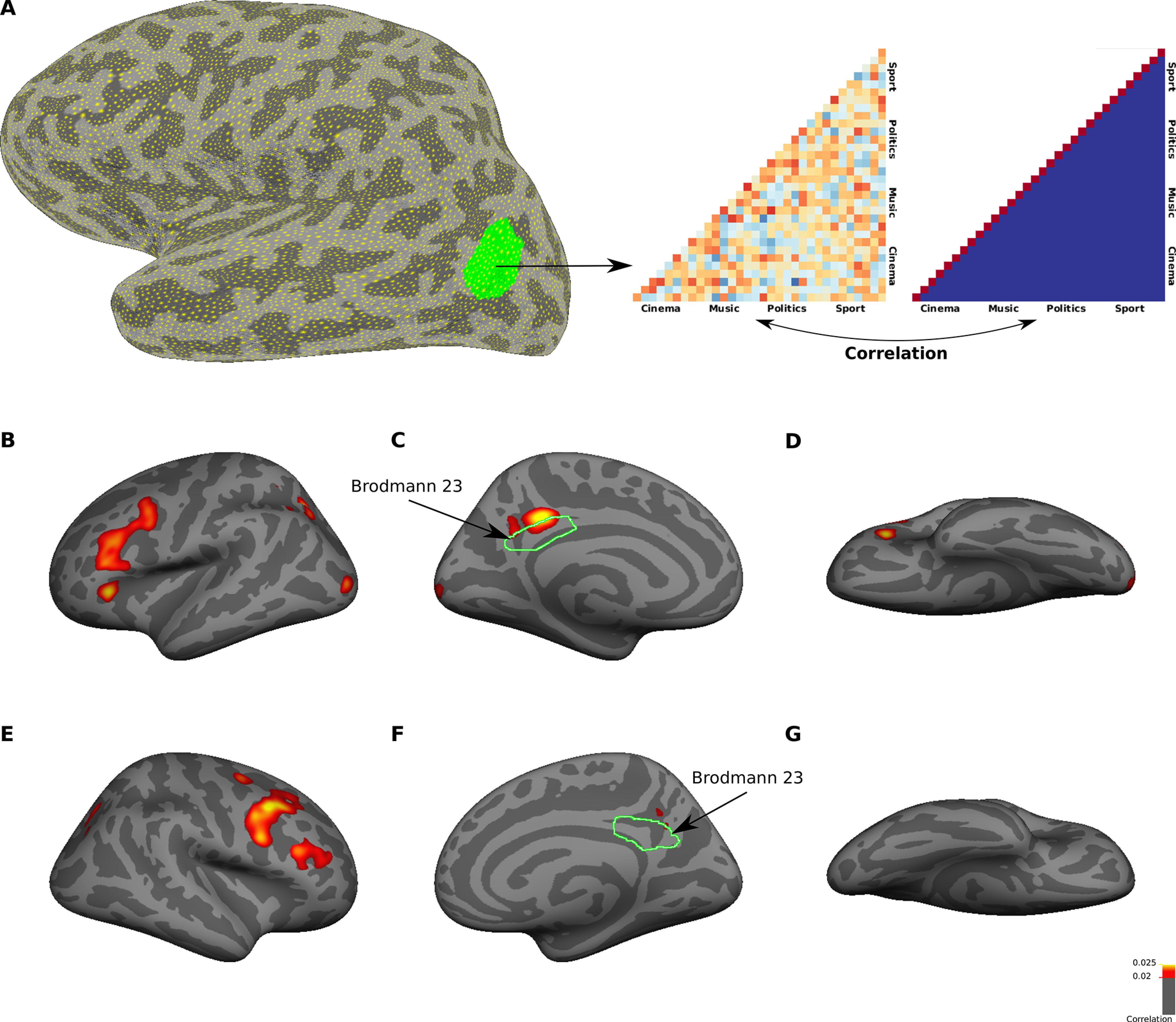
Whole-brain searchlight analysis for identifying regions that encode familiar face identities. ***A***, The procedure for surface-based searchlight analysis was similar to the one described in Figure 7*A*, except that here we used a model to test pattern information for familiar face identities. ***B–G***, The maps show averaged correlation coefficient values (Kendall's tau correlation) from 16 subjects in left (***B–D***) and right (***E–G***) hemispheres. Only vertices with a significant (*p* < 0.05, *t* test, uncorrected) correlation are included in the maps. The correlation maps are displayed on lateral (***B***, ***E***), medial (***C***, ***F***), and ventral (***D***, ***G***) views of inflated fsaverage surface.

To localize regions containing information for familiar face identities, we did a surface-based searchlight analysis (Fig. 10*A*). For each region on the cortical surface, sdRSM with the size 32 × 32 was computed. Rows and columns of the matrix corresponded to 32 individual examples of familiar faces. Neural sdRSM was correlated with identity matrix of the same size using rank correlation, then the correlation coefficient values were mapped on the cortical surface. The searchlight maps from group analysis are shown on the inflated surfaces of both hemispheres in Figure 10*B–G*. Different identities of familiar faces could be decoded significantly in PCC of left hemisphere, in a region that was located immediately superior to Brodmann area 23. Information for familiar face identities was also represented in discrete regions within LPFC of both hemispheres. In the searchlight analysis for familiar face identities, the correlation values were low, possibly because of a high number of conditions used in this analysis. Nonetheless, the searchlight maps revealed localized regions which had significant information for familiar face identities. Similar to the result of searchlight analysis for familiar face subcategories, no significant cluster was found in ventral temporal cortex.

### Topographic organization of semantic face representations in PCC

**Figure 11.**
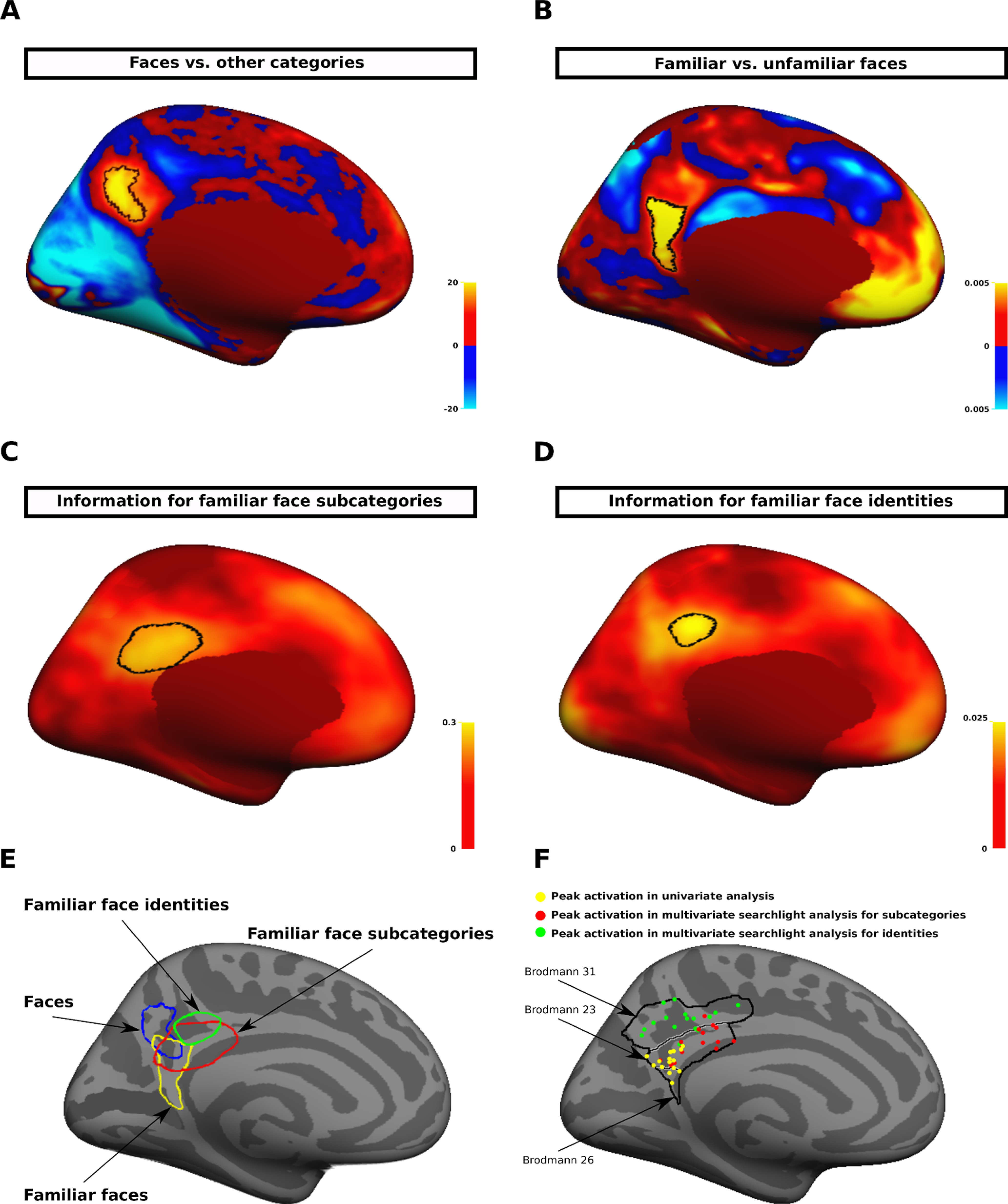
Topographic organization of semantic face representations in PCC. ***A***, Unthresholded map of group-average (*N* = 787 subjects) activation for the contrast of faces (red/yellow) versus other categories (blue/cyan) from the HCP database. The most significant face-selective vertices (the top 1% of vertices with the highest z values) are indicated with a black border. The color scale bar represents z values. ***B***, Unthresholded map of group-average activation for the contrast of familiar (red/yellow) versus unfamiliar (blue/cyan) faces. The significant vertices (*p* < 0.05, *t* test, FDR-corrected) are indicated with a black border. The color scale bar represents *p* values. ***C***, Unthresholded map of searchlight analysis for subcategories of familiar faces. The area within the black border indicates the thresholded vertices (Kendall's tau correlation > 0.2, *p* < 0.05, *t* test, FDR-corrected) shown in Figure 7*C*. The color scale bar is truncated to show positive correlation values. ***D***, Unthresholded map of searchlight analysis for identities of familiar faces. The area within the black border indicates the thresholded vertices (Kendall's tau correlation > 0.02, *p* < 0.05, *t* test, uncorrected) shown in Figure 10*C*. The color scale bar is truncated to show positive correlation values. ***E***, Topological organization of areas representing information of faces (blue), familiar faces (yellow), subcategories of familiar faces (red), and identities of familiar faces (green) in left PCC. ***F***, The locations of peak activations for univariate analysis (yellow dots), multivariate subcategory analysis (red dots), and multivariate identity analysis (green dots) within a merged ROI of Brodmann areas 23, 26, and 31 in the left hemisphere. Each colored dot corresponds to one subject. One subject in the univariate analysis and one subject in the multivariate subcategory analysis did not have a cluster of activation within the ROI. All maps in this figure are displayed on medial view of the left hemisphere in fsaverage space.

A consistent feature of our univariate and multivariate analyses was an activation in PCC/precuneus of left hemisphere. However, the peaks of activations for different levels of semantic face processing appeared to be located in different spots within PCC. In the next analysis, we aimed to investigate the topographic relationship between representations for familiar faces, familiar face subcategories, and familiar face identities in left PCC. In this analysis, we also included a map from the HCP database, which showed visual activations for faces in left PCC. This map was obtained by comparing faces versus other categories (places, objects, and body parts). Figure 11*A–D* show unthresholded maps from four analyses: (1) univariate comparison between faces versus other categories; (2) univariate comparison between familiar faces versus unfamiliar faces; (3) multivariate searchlight analysis for familiar face subcategories; and (4) multivariate searchlight analysis for familiar face identities. In each map, the “hot spot” area, which was located in left PCC, was delineated. These hot spot areas overlapped with each other (Table 1), although there was also a systematic shift in the location of areas (Fig. 11*E*). The area distinguishing faces from other categories was located in the posterior-superior subdivision of left PCC. The area distinguishing familiar faces from unfamiliar faces was located in the posterior-inferior subdivision of left PCC. Finally, the area distinguishing different subcategories and identities of familiar faces was located more anteriorly in left PCC.

**Table 1.** Spatial overlap between the representations of faces, familiar faces, familiar face subcategories, and familiar face identities in PCC

	Faces	Familiar faces	Familiar face subcategories	Familiar face identities
Faces	1	0.32	0.23	0
Familiar faces	0.32	1	0.4	0.01
Familiar face subcategories	0.23	0.4	1	0.38
Familiar face identities	0	0.01	0.38	1

The spatial overlap was quantified using dice coefficient (1: full overlap, 0: no overlap).

To evaluate the topographic relationship between representations for familiar faces, familiar face subcategories, and familiar face identities at the individual subject level, we obtained the peaks of clusters of activations for univariate analysis (the highest significant response to familiar than unfamiliar faces) and multivariate searchlight analyses (the highest correlation between response pattern and categorical model) in each subject. The peak-finding was confined to the ROIs of Brodmann areas 23, 26, and 31 in the left hemisphere. As shown in Figure 11*F*, the peak activations for subcategory analysis were shifted anteriorly, and the peak activations for identity analysis were shifted superiorly. To test whether these shifts were statistically significant, two (one-component and three-component) 3-D Gaussian mixture models were fitted to the distribution of peak activations. Each k-component model was defined as a linear combination of k Gaussian distributions. The parameters of Gaussian distributions including means, covariance matrices, and mixing proportions were explicitly specified using information of each group. To choose the model that better explains the distribution of peak activations, we evaluated each model's goodness-of-fit using the Akaike information criterion (AIC) statistic. The AIC statistics derived from one-component and three-component model fits were 870.53 and 745.73, respectively. The three-component model appeared to be significantly preferred using the relative likelihood estimation (Wagenmakers and Farrell, 2004). For all pairs of groups, the two-component model also had significantly better fit compared with the one-component model.

**Figure 12.**
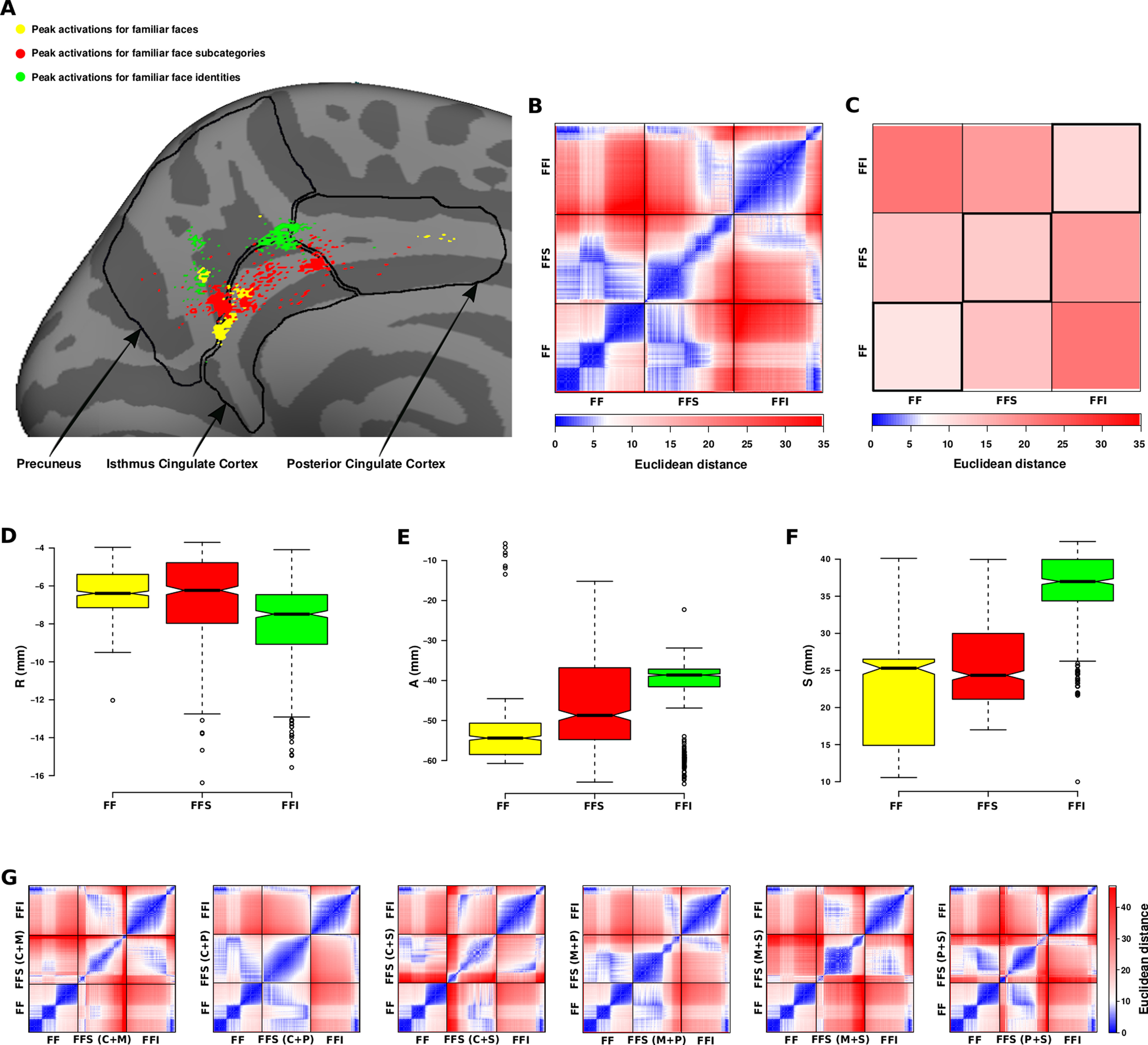
Quantitative analysis for evaluating the topographic relationship between representations of familiar faces, familiar face subcategories, and familiar face identities in PCC. ***A***, Partial medial surface of the left hemisphere including three anatomic regions: isthmus cingulate cortex, precuneus, and PCC. Distributions of dots correspond to the peak activations from univariate analysis for familiar faces (FF; yellow), multivariate searchlight analysis for familiar face subcategories (FFS; red), and multivariate searchlight analysis for familiar face identities (FFI; green). To obtain each peak activation, data from a specific combination of runs were analyzed, then the responses were averaged across 16 subjects. In total, 500 peak activations were obtained for each type of face representation. ***B***, A dissimilarity matrix (1500 × 1500) representing topological dissimilarities of three distributions of peaks (FF, FFS, and FFI) using Euclidean distance measure. Each element of the matrix corresponds to the pairwise distance of peak locations. To plot the distance values, anti-Robinson seriation by simulated annealing (ARSA) method (Brusco et al., 2008) was used. ***C***, A dissimilarity matrix (3 × 3) representing average Euclidean distances between three distributions of peaks. The color bars in ***B***, ***C*** indicate Euclidean distance values in mm. ***D–F***, The boxplots show distributions of peak locations for FF (yellow), FFS (red), and FFI (green) along each axis of the RAS coordinate system. The R, A, and S values are in mm, and they indicate the location of points relative to a reference point along right, anterior, and superior directions. The reference point of the RAS coordinate system is centered in the voxel whose volume index is (128, 128, 128). The summary statistics shown in the boxplots include lower and upper quartiles of distributions (bottom and top of the boxes), the median (center line), the range within 150% of the interquartile interval (whiskers), and outliers (circles). ***G***, Six versions of dissimilarity matrices (1500 × 1500) in which the peak activations for familiar face subcategories were obtained by including two (out of four) subcategories in the analysis.

To quantify topographic relationship between three levels of semantic face representations, we first estimated distributions of peak activations for familiar faces, familiar face subcategories, and familiar face identities (Fig. 12*A*). Each peak activation was obtained by analyzing a specific combination of runs (for more details, see Materials and Methods). Then, we extracted the spatial coordinates of the peaks (vertices) within the White surface RAS coordinate system and compared the spatial distributions of peaks simultaneously (Fig. 12*D–F*). The result of statistical analysis revealed that the spatial difference between distributions was highly significant (Kruskal–Wallis test, df = 6, *p* ≪ 0.05). Pairwise comparisons also showed that each of the distributions was spatially distinct from the other two distributions (two-tailed Mann–Whitney *U* test, *p* ≪ 0.05, adjusted for ties, Bonferroni-corrected). This distinction was also evident when pairwise comparisons were performed along each axis (R, A, and S) separately (two-tailed Mann–Whitney *U* test, *p* ≪ 0.05, adjusted for ties, Bonferroni-corrected). Considering the voxel size in our fMRI data acquisition (3.5 × 3.5 × 3.5 mm), the median of peak distribution for familiar faces was located two to three voxels posterior to the median of peak distribution for familiar face subcategories and three to four voxels posterior to the median of peak distribution for familiar face identities. Moreover, the median of peak distributions for familiar faces and familiar face subcategories was located three to four voxels inferior to the median of peak distribution for familiar face identities.

In another analysis, we computed a dissimilarity matrix (1500 × 1500) and an average dissimilarity matrix (3 × 3) for three spatial distributions of the peaks using Euclidean distance measure (Fig. 12*B*,*C*). Using ANOSIM analysis, dissimilarity of three distributions (between-group distances minus within-group distances) was quantified. Consistent with previous results, all three spatial distributions were significantly distinct from each other (*R* = 0.3036, *p* < 0.001). In a *post hoc* test, differences between all pairs of distributions were significant (all *p* < 0.003, Bonferroni-corrected). In all cases, the average within-group distance was lower than the average between-group distance (Fig. 12*C*). To evaluate the reliability of effects, we repeated the multivariate searchlight analysis six times, each with one pair (out of six possible pairs) of familiar face subcategories (cinema+music, cinema+politics, and so on). For each pair, 500 peaks of activation were obtained. Again, the ANOSIM analysis revealed a significant difference between distribution of peaks for each pair of familiar face subcategories and distributions of peaks for familiar faces and familiar face identities (all *p* < 0.006, Bonferroni-corrected; Fig. 12*G*). Thus, the observed shift in the location of activations for familiar face subcategories was a reliable effect, not induced by a specific subcategory of faces.

### Functional connectivity of semantic face areas in PCC

**Figure 13.**
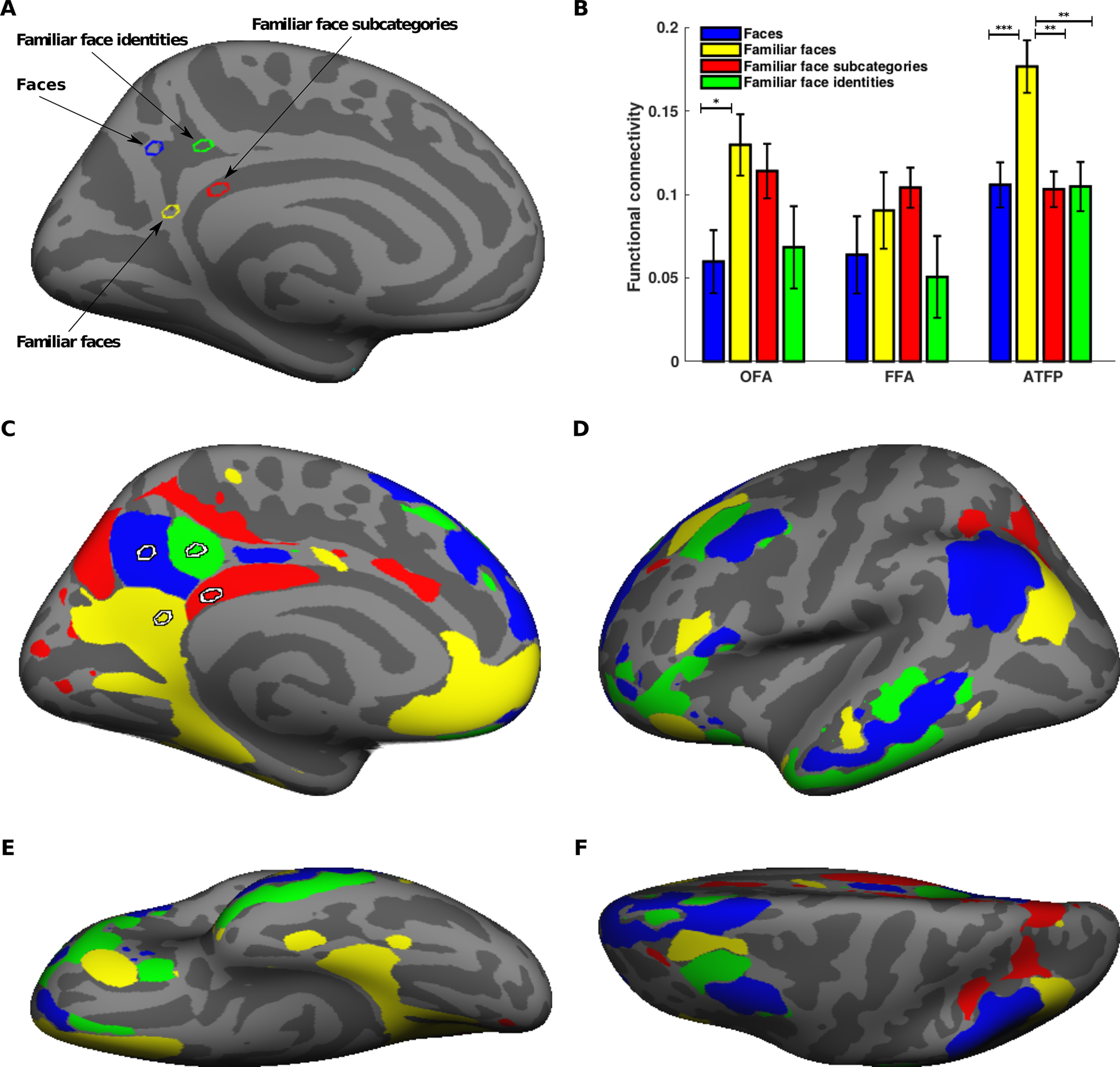
Whole-brain functional connectivity patterns of semantic face areas in PCC. ***A***, The location of four seed regions corresponding to the representation of faces (blue), familiar faces (yellow), familiar face subcategories (red), and familiar face identities (green). The seed regions are displayed on the medial view of inflated cortex in left hemisphere. ***B***, The bar plot shows the amount of ROI-based functional connectivity (Pearson correlation) between the seed regions of left PCC and the face-selective areas of ventral temporal cortex (OFA, FFA, and ATFP). OFA, FFA, and ATFP were localized in the left hemisphere using our functional localizer. Error bars denote the SEM calculated across subjects. Asterisks indicate significant differences between conditions (*p < 0.05, **p < 0.005, ***p < 0.0005, one-way repeated measures ANOVA with Tukey–Kramer *post hoc* test). ***C–F***, The result of the winner-take-all functional connectivity analysis. The maps are displayed on the medial (***C***), lateral (***D***), ventral (***E***), and dorsal (***F***) views of inflated cortex in left hemisphere. The colors in the maps correspond to the color of seed regions in panel ***A***.

To evaluate the pattern of functional connectivity between the semantic face areas of left PCC and the face-selective areas of ventral temporal cortex (OFA, FFA, and ATFP), we first defined four seed regions corresponding to the representation of faces, familiar faces, familiar face subcategories, and familiar face identities in left PCC (Fig. 13*A*). Then, using an ROI-based functional connectivity analysis, we calculated the magnitude of functional coupling between seed regions and face-selective ROIs in ventral temporal cortex (Fig. 13*B*). The results showed that among the PCC regions, the region representing information of familiar faces had the strongest functional coupling with ATFP. The coupling of this region with ATFP was significantly stronger than the coupling of other PCC regions with ATFP (*F*_(3,42)_ = 3.99, *p* < 0.05, one-way repeated measures ANOVA).

To test whether the PCC regions were functionally connected to distinct areas in other parts of cortex, we performed a whole-brain vertex-wise functional connectivity analysis. The PCC regions showed strong functional coupling with areas within temporo-parietal cortex, lateral temporal cortex, and superior frontal cortex (Fig. 13*C–F*). Importantly, the functional connectivity patterns were topographically organized, meaning that each PCC region was preferentially connected to a specific subdivision of temporo-parietal, lateral temporal, and superior frontal areas. These subdivisions were topographically adjacent to each other. Additionally, consistent with the results of ROI-based analysis, ventral temporal areas of cortex were preferentially connected to the PCC region representing information of familiar faces.

## Discussion

In this study, we investigated how different levels of semantic information for familiar faces were represented in the brain. To address this question, we tested the brain responses to the images of familiar and unfamiliar faces. Familiar faces were selected from four categories of famous people, each with specific social information and semantic associations. To discover cortical areas encoding semantic information of faces, we gradually increased the specificity of our fMRI analysis, from univariate analysis to different levels of multivariate analysis. The result of univariate analysis showed that information about familiarity of faces was represented in PCC, ACC, ATL, and TPJ. This result was consistent with previous neuroimaging findings (Gobbini et al., 2004). The result of multivariate searchlight analysis for familiar face subcategories revealed that information about different subcategories could be decoded from neural patterns in PCC and also regions within the frontoparietal network. The result of multivariate searchlight analysis for familiar face identities revealed that information about different identities could be decoded from neural patterns in PCC and also areas in the frontal cortex. Identification of individual faces of familiar face category could be considered the most specific level of semantic face processing. To include a more general level of semantic face processing, we used the HCP database with a large sample of subjects to localize neural structures that were more sensitive to faces than other semantic categories such as places, objects, and body parts. As expected, these structures included face patches within ventral temporal cortex plus a relatively new area within PCC/precuneus. The activations in PCC/precuneus from all levels of analyses had some overlap with each other. However, the peak activations were spatially distinct. Overall, these results demonstrated that different subdivisions within PCC/precuneus may have a central role in processing the face-related semantic information. Moreover, semantic information of faces appeared to be progressively disentangled/abstracted by a network of spatially adjacent areas in PCC/precuneus. Semantic processing of faces in this network could be complimentary to perceptual processing of faces, which is mainly mediated by the face-selective network in ventral temporal cortex.

### Representation of information for familiar faces

In humans, PCC/precuneus (as part of the posterior medial cortex) plays an important role in representing face-related semantic information. Neuroimaging studies have reported that PCC/precuneus is well activated by familiar faces including one's own face (Kircher et al., 2000; Platek et al., 2008; Sugiura, 2015), personally familiar faces (Gobbini et al., 2004; Leibenluft et al., 2004; Pierce et al., 2004; Sugiura et al., 2009; Lee et al., 2013; Blank et al., 2014), and famous faces (Leveroni et al., 2000; Bernard et al., 2004; Eger et al., 2005). Neuropsychological evidence also provides support for the role of PCC/precuneus in familiar face processing. One study implicated that failure of familiar/famous face recognition in congenital prosopagnosic patients could be attributed to abnormal functioning of PCC/precuneus in the extended face network (Avidan and Behrmann, 2009). Another study showed that abnormal behavior of autistic subjects in recognizing familiar faces was correlated to the lower extent of activity in this area (Pierce et al., 2004).

While successful recognition of familiar faces entails both visual component and personal knowledge, the activity within PCC/precuneus was mostly attached to person knowledge. The role of this area in person knowledge retrieval was corroborated by studies that experimentally associated faces with fictional knowledge (Todorov et al., 2007; Cloutier et al., 2011) and studies that attributed the higher extent of activities for personally familiar than famous faces to the higher amount of associated person knowledge, either episodic (Gobbini et al., 2004) or semantic (Fairhall et al., 2014) knowledge. Other studies showed that the higher sensitivity to personally familiar than famous faces in PCC/precuneus was mediated by the caudal part of this area, and the activation relevant to famous faces was located more rostrally (Sugiura et al., 2009; Blank et al., 2014). In the present study, the location of familiarity-selective area in PCC/precuneus was consistent with the location that was reported to be selective for famous faces (i.e., posterior to corpus callosum).

PCC was not the only region that revealed higher activity to familiar faces. Consistent with previous findings (Leveroni et al., 2000; Gobbini and Haxby, 2007; Visconti Di Oleggio Castello et al., 2017), other areas in ACC, ATL, and TPJ also revealed sensitivity to familiar stimuli. It has been suggested that these areas are recruited in processes such as person perception and person knowledge. Here, while these areas showed a strong activity with respect to the familiarity attribute, they did not show any representation of categorical information for subcategories of familiar faces. Thus, the observed activity in these areas was less likely to be related to contextual, social, and semantic information of faces. The activity in ACC may have been driven by information about traits of familiar faces, which could have been retrieved automatically by subjects. Activation in ACC has been reported in tasks requiring participants to form imressions about others, make inferences about psychological traits of others (Jenkins et al., 2008; Denny et al., 2012; Wagner et al., 2018), make inferences about mental states of others (Frith and Frith, 1999), and unconsciously process emotional/affective information of faces (Killgore and Yurgelun-Todd, 2004). The activity in TPJ could also reflect covert retrieval of distinct names associated with familiar faces (Gesierich et al., 2012).

### Representation of information for subcategories of familiar faces

In the multivariate analysis, we found an area within PCC, which contained reliable and decodable information for familiar face subcategories. Functionally, PCC/precuneus is typically involved in high-level cognitive processes including episodic and semantic memory. The posterior neural structures within PCC/precuneus were reported to be sensitive to autobiographical retrieval and episodic memory, whereas the anterior neural structures within this area were more responsive to semantic information (Cavanna and Trimble, 2006; Binder et al., 2009). What we know about the function of PCC is largely based on the cognitive tasks which differentiate neural activities during episodic versus semantic information processing. However, it is not clear how various types of episodic or semantic information are represented in PCC. One possibility is that this area has only a modulatory role in encoding these information, which happens through an enhanced activity during episodic/semantic functional tasks. Alternatively, information of different episodic/semantic attributes may be represented in the patterns of activity in this area. Our analysis showed that information of four categories of famous faces could be decoded from the patterns of activity in PCC. Since knowledge about famous people is usually acquired through semantic information (not through direct social relationships and episodic events), we can suggest that PCC plays an important role in encoding different attributes of semantic information.

In our analysis to define regions representing information for subcategories of familiar faces, we also found activities in a network of areas within PPC and LPFC. Although activity in these areas has been reported invariably during tasks involving semantic processing (Fletcher and Henson, 2001) or maintenance of semantic information (Gabrieli et al., 1998), recruitment of these areas might be less bound to long-term neural representation of semantic information. There are numerous evidence showing that a distributed network of areas in parietal cortex and PFC, located at the interface of sensory and motor systems, gradually transforms stimulus-related information to an appropriate behavioral response (Christophel et al., 2017). PPC has been reported to be involved in short-term memory representation of task-related visual features (like color and shape; Marois and Todd, 2004; Xu and Chun, 2006; Bettencourt and Xu, 2016) and behaviorally relevant object identity information (like famous faces and well-known cars; Jeong and Xu, 2016). Prefrontal regions are also engaged in encoding short-term memories of sensory stimuli in an amodal format for the guidance of upcoming behavioral actions (Spitzer et al., 2014). In our experiment, subjects were asked to attend to the category of the presented face stimulus, and after a delay (1 s), compare it with a letter stimulus and report a match/non-match response. It is possible that semantic information of face categories is initially processed in PCC, then the categorical information is sent to the network of parietofrontal areas where the attended information is temporarily maintained in a short-term memory buffer for an upcoming comparison with the letter stimulus.

### Representation of information for familiar face identities

In the multivariate analysis, we found a distinct area within PCC, which contained reliable and decodable information for familiar face identities. The information in this area was weak; however, the result was consistent with the finding of a recent study which reported decodable information for familiar face identities in the dorsal part of PCC (Visconti Di Oleggio Castello et al., 2017). Several factors may have contributed to weak identity-specific information. As mentioned in Results, the searchlight analysis for familiar face identities had a high number of conditions and consequently a low statistical power. There was also a low demand for identification in our task, as subjects were required to attend to semantic subcategories (not specific identities) of familiar face stimuli (e.g., a politician face instead of Barak Obama's face). An fMRI study with an optimized design for familiar face identification may show strong activities in PCC for different face identities.

In addition to PCC, we found that regions within LPFC also contained information for familiar face identities. These prefrontal representations were slightly stronger in the right hemisphere. A recent study has reported an area in a similar location in the right inferior frontal cortex for decoding visually-familiar individual faces invariant of head view (Guntupalli et al., 2017). View-invariant representation of face identities in this area might be critical for engaging PCC and other parts of the extended face network in the successful recognition of familiar individuals.

### Representation of perceptual information for faces in PCC

In the comparison between faces versus other object categories (a more general level of face processing), we localized a face-selective area in the posterior part of PCC. Given the fact that stimuli used in this level of analysis were all unfamiliar and did not contain any predetermined personal knowledge, face representation in this area would not be related to attributes such as familiarity or knowledge about each identity. Rather, the area may encode some aspects of perceptual information for faces. This idea is supported by studies which showed the role of this area in perceptual learning of faces and also in the conditions of increased perceptual demands when participants had to access to the information of specific identities. Neuroimaging studies reported that this area was face-responsive using perceptual learning paradigm (Katanoda et al., 2000; Leveroni et al., 2000; Maguire et al., 2001; Kosaka et al., 2003; Gobbini and Haxby, 2007; Cloutier et al., 2011; Natu and O'Toole, 2015; Anzellotti and Caramazza, 2016), fMR-adaptation paradigm (Pourtois et al., 2005; Weibert et al., 2016), visual imagery (Ishai et al., 2000), functional localizers with dynamic facial stimuli (Fox et al., 2009), and functional localizers with a working memory task (Anzellotti and Caramazza, 2017). A recent EEG-fMRI study also showed that activation in PCC/precuneus covaried with early ERP markers of face processing (Bayer et al., 2018). In our analysis, we used “category localizer” data from the HCP database. A large sample size and presence of a working memory task in this dataset enabled us to detect a robust and localized activity for faces in PCC.

### Topographic organization of semantic face representations in PCC

Within PCC, the peak activations for different semantic face representations were spatially distinct, although the extent of activations showed some overlap. Areas representing faces and familiarity were located in the posterior part of PCC, whereas areas representing subcategories and identities of familiar faces were located in the anterior part of PCC. This posterior to anterior gradient of face representations in medial parietal cortex is analogous to the posterior-anterior organization of face-selective areas in ventral temporal cortex (i.e., OFA -> FFA -> ATFP). The “ventral pathway” and “medial pathway” of face processing may serve as two parallel pathways with complementary functions: the ventral pathway for perceptual processing of faces (analysis of facial features, configuration, viewpoint, and identity) and the medial pathway for semantic processing of faces (detailed analysis of familiar faces). Although, using fMRI, we did not (and perhaps we could not) investigate the connectivity between different subdivisions of PCC, we speculate that there is a flow of face-related information from posterior to anterior PCC, in which semantic information is processed at multiple levels, from general to more specific levels. Such posterior-anterior distinction has been also proposed for a nearby region in retrosplenial complex (RSC) which is involved in scene perception and spatial navigation (Silson et al., 2016). It has been shown that the posterior part of RSC processes scene-related visual information, whereas the anterior part of RSC processes mnemonic information to reconstruct scenes from memory (Silson et al., 2019a). In medial parietal cortex, the area recruited during memory recall of specific places is located posterior and ventral to the area recruited during memory recall of familiar people (Silson et al., 2019b).

### Functional connectivity of semantic face areas in PCC

In the whole-brain functional connectivity analysis, we observed that while the semantic face areas of PCC were highly interconnected, each of these areas had a distinct pattern of connectivity with other parts of cortex. This result is at odds with the earlier reports which considered PCC as a homogeneous and single hub of DMN (Buckner et al., 2008). Instead, our finding is consistent with the idea that DMN includes multiple subnetworks (Yeo et al., 2011; Andrews-Hanna et al., 2014; Chiou et al., 2020), and therefore, the ventral-dorsal or posterior-anterior subdivisions of PCC show distinct patterns of connectivity (Vogt et al., 2006; Margulies et al., 2009; Cauda et al., 2010; Leech et al., 2011; Bzdok et al., 2015). More specifically, the ventral-posterior subdivision of PCC, which represented information of familiar faces, was prominently connected to the parahippocampal cortex and structures of MTL. This pattern of connectivity was similarly reported in the previous studies of resting-state functional connectivity (Bzdok et al., 2015) and anatomic axonal tracing (Kobayashi and Amaral, 2003, 2007). The observed connectivity pattern corroborates the role of this region in subserving episodic/semantic memory retrieval (Cavanna and Trimble, 2006).

The ventral-posterior and dorsal-posterior subdivisions of PCC were distinctly connected to the ventral and dorsal subdivisions of temporo-parietal cortex, respectively. This topographic connectivity suggests a strong functional coupling between PCC and temporo-parietal cortex, which might be crucial for semantic face processing.

A recent study reported that a face-selective cluster in PCC (in medial parietal cortex) was strongly connected to the lateral part of fusiform gyrus including FFA (Silson et al., 2019b). This study also suggested that the ventral/dorsal subdivisions of medial parietal cortex, representing information of scenes/faces, were preferentially connected to the lateral/medial subdivisions of fusiform gyrus. However in our analysis, we could not detect any strong functional couplings between lateral subdivision of fusiform gyrus (including FFA) and the semantic face areas of PCC. Rather, we found strong functional coupling between ATFP and a PCC area representing information of familiar faces.

### The role of OFA and FFA in semantic face processing

There has been a considerable controversy surrounding sensitivity of core face-selective areas (OFA and FFA) to semantic information of familiar faces. Some studies reported no significant modulation of activity for the familiarity attribute (Dubois et al., 1999; Leveroni et al., 2000; Gorno-Tempini and Price, 2001; Eger et al., 2005; Ramon et al., 2015), while others found weak results (Sergent et al., 1992; Kosaka et al., 2003; Rossion et al., 2003; Gobbini and Haxby, 2006). One potential caveat of the studies, which reported high-level abstract information of faces in core face-selective areas, was that the stimuli used in those studies were not carefully matched based on low-level visual features. To control for this confound, one study compared semantic component of faces when the visual information of faces was absent (van den Hurk et al., 2011). The results revealed weak selectivity to semantic information in FFA. Our univariate analysis showed no sensitivity to familiarity in OFA and FFA. Additionally, our multivariate analysis showed that these areas could not discriminate between patterns of activities for different subcategories of familiar faces. Overall, these findings support the idea that core face-selective areas are not particularly involved in processing the semantic information of faces, at least at the macroscopic level of fMRI.

### The role of ATL in semantic face processing

While PCC was sensitive to different levels of semantic information of faces, no such sensitivity was observed in ATL. It is generally accepted that ATL plays a critical role in semantic processing (Skipper et al., 2011), and its ventral subdivision contains view-invariant information of face identities (Kriegeskorte et al., 2007; Nestor et al., 2011; Anzellotti et al., 2014). It has been suggested that face-selective areas in ATL might link invariant perceptual information of faces with person-specific semantic information (Collins and Olson, 2014). A recent fMRI study in monkeys also reported two face patches in macaque ATL, which responded selectively to faces of familiar monkeys (Landi and Freiwald, 2017). In our study, we used famous faces which clearly contained semantic information; however, we could not observe any categorical information for famous faces in face-selective areas of ATL. Similarly another fMRI study failed to find successful decoding of the identity of famous faces in ATL (Axelrod and Yovel, 2015). ATL is located in a region which is prone to susceptibility artifacts (Devlin et al., 2000; Gorno-Tempini et al., 2002). Thus, the lack of pattern information in this region could potentially be because of insufficient signal-to-noise ratio of fMRI signal. It is also possible that neural representations of semantic information in ATL are based on a sparse code which may not be resolvable at current imaging resolutions. As shown previously, sparsely distributed neurons in anterior medial temporal cortex could encode abstract information of faces (e.g., a neuron that responded selectively and invariantly to pictures and word of a famous celebrity like Jennifer Aniston; Quian Quiroga et al., 2005). Further research using optimized imaging techniques could shed light on whether face-selective areas within ATL contain categorical information for familiar faces.

### Comparison of familiar face processing in humans and monkeys

Using fMRI, one study investigated familiar face processing in monkeys and reported two areas in macaque ATL, which responded selectively to faces of familiar monkeys (Landi and Freiwald, 2017). This study did not find activations in macaque PCC (personal communication). However, based on resting-state fMRI data, the medial parietal regions in monkeys have been shown to be functionally coupled to the core face processing areas (Schwiedrzik et al., 2015).

In addition to detecting familiarity, human PCC is more broadly involved in semantic processing. One aspect of this processing is to represent contextual information associated with people. It is possible that semantic associations are generally weak in monkeys, and to activate these associations in macaque PCC, one may need to use more sophisticated stimuli such as dynamic video clips of personally familiar monkeys interacting in a naturalistic situation.

In conclusion, collectively, we found distinct areas within PCC/precuneus, which represented semantic information of faces in different levels of abstraction. The functional segregation of neural structures within PCC suggests that semantic information of familiar faces could be progressively disentangled by a network of hierarchically organized areas, a network which is dissociable from the network of classic face-selective areas in ventral temporal cortex. In our study, we used a limited number of face subcategories and individual faces. fMRI responses in the PCC areas could be tested for other social classes of face stimuli and also for a larger number of face identities. Furthermore, it would be interesting to evaluate cortical representations for subcategories of familiar faces, familiar scenes/buildings, and familiar objects in one experimental setup. Such study could provide a more comprehensive view of functional organization in medial parietal cortex.
